# Crosstalk of HDAC4, PP1, and GSDMD in controlling pyroptosis

**DOI:** 10.1038/s41419-024-06505-z

**Published:** 2024-02-07

**Authors:** Weilv Xu, Qiao Jin, Xinyue Li, Danyue Li, Xinyu Fu, Nan Chen, Qian Lv, Yuhua Shi, Suhui He, Lu Dong, Yang Yang, Yuqi Yan, Fushan Shi

**Affiliations:** 1 Key Laboratory of Animal Virology of Ministry of Agriculture, Center for Veterinary Sciences, Zhejiang University, Hangzhou, Zhejiang China; 2 Department of Veterinary Medicine, College of Animal Sciences, Zhejiang University, Hangzhou, Zhejiang China; 3 Zhejiang Provincial Key Laboratory of Preventive Veterinary Medicine, Zhejiang University, Hangzhou, Zhejiang China; 4 Key Laboratory of Applied Technology on Green-Eco-Healthy Animal Husbandry of Zhejiang Province, Zhejiang Provincial Engineering Research Center for Animal Health Diagnostics & Advanced Technology, Zhejiang International Science and Technology Cooperation Base for Veterinary Medicine and Health Management, China-Australia Joint Laboratory for Animal Health Big Data Analytics, College of Animal Science and Technology & College of Veterinary Medicine of Zhejiang A&F University, Hangzhou, Zhejiang China

**Keywords:** Cell death and immune response, Inflammasome

## Abstract

Gasdermin D (GSDMD) functions as a pivotal executor of pyroptosis, eliciting cytokine secretion following cleavage by inflammatory caspases. However, the role of posttranslational modifications (PTMs) in GSDMD-mediated pyroptosis remains largely unexplored. In this study, we demonstrate that GSDMD can undergo acetylation at the Lysine 248 residue, and this acetylation enhances pyroptosis. We identify histone deacetylase 4 (HDAC4) as the specific deacetylase responsible for mediating GSDMD deacetylation, leading to the inhibition of pyroptosis both in vitro and in vivo. Deacetylation of GSDMD impairs its ubiquitination, resulting in the inhibition of pyroptosis. Intriguingly, phosphorylation of HDAC4 emerges as a critical regulatory mechanism promoting its ability to deacetylate GSDMD and suppress GSDMD-mediated pyroptosis. Additionally, we implicate Protein phosphatase 1 (PP1) catalytic subunits (PP1α and PP1γ) in the dephosphorylation of HDAC4, thereby nullifying its deacetylase activity on GSDMD. This study reveals a complex regulatory network involving HDAC4, PP1, and GSDMD. These findings provide valuable insights into the interplay among acetylation, ubiquitination, and phosphorylation in the regulation of pyroptosis, offering potential targets for further investigation in the field of inflammatory cell death.

## Introduction

Pyroptosis is a type of programmed necrotic cell death, featuring cell swelling, and large bubbles blowing from the plasma membrane, and has received increasing attention for its association with innate immunity [1–3]. Pyroptosis is mediated by inflammasomes assembly and activation [4, 5]. Canonical inflammasomes, such as NLRP1, NLRP3, NLRC4, and AIM2 inflammasomes, assemble in the cytosol to recruit and activate caspase-1. Noncanonical inflammasomes are activated by human caspase-4/5 (mouse orthologs caspase-11) directly binding to intracellular lipopolysaccharide (LPS) [5–8].

Gasdermins are reported to be the executioner of pyroptosis. The gasdermin family consists of Gasdermin A-E and DFNB59, all of which exhibit propyroptotic activity and possess an autoinhibited structure [1–3, 9]. Among these members, Gasdermin D has been the subject of extensive investigation. GSDMD can undergo cleavage by caspase-1/4/5/11, resulting in the release of its N-terminal domain (GSDMD-p30). This domain has the ability to oligomerize, forming pores in the cell membrane that lead to the release of inflammatory cytokines and the initiation of pyroptosis [10–13].

Recently, posttranslational modifications (PTMs) have gained prominence as pivotal regulators of pyroptosis, exerting influence on various components of inflammasomes. Specifically, SIRT2-mediated deacetylation of NLRP3 has the capacity to render the NLRP3 inflammasome inactive, whereas SIRT3 deacetylates NLRC4, fostering the activation of the NLRC4 inflammasome [14, 15]. Additionally, ubiquitination has been shown to modify the function of GSDMD in pyroptosis. Our previous research demonstrated that SYVN1 mediates K27-linked polyubiquitination of GSDMD to promote the pyroptotic cell death [16]. Moreover, Shigella ubiquitin ligase IpaH7.8 utilizes ubiquitination to induce GSDMD degradation, thereby preventing pyroptosis [17]. Furthermore, there exists a connection between different posttranslational modifications. Acetylation of OTUD3 significantly enhances its activity in forming Lys63 linkages on MAVS, and acetylation can promote ubiquitination of p62 [18, 19]. Nonetheless, there has been limited research directly exploring the post-translational modifications of GSDMD, necessitating further clarification on the relationship between these post-translational modifications and GSDMD.

In this study, we report that GSDMD could be acetylated and histone deacetylase 4 (HDAC4) inhibits pyroptosis through deacetylating GSDMD. Moreover, we identify the Protein phosphatase 1 (PP1) as the phosphatase for HDAC4. The phosphorylation of HDAC4 exhibits significant effects on its ability to suppress pyroptosis. Interestingly, we discover that the acetylation of GSDMD promotes its ubiquitination, a mechanism not previously described in relation to pyroptosis.

## Results

### Acetylation of GSDMD promotes pyroptosis

**Fig. 1:**
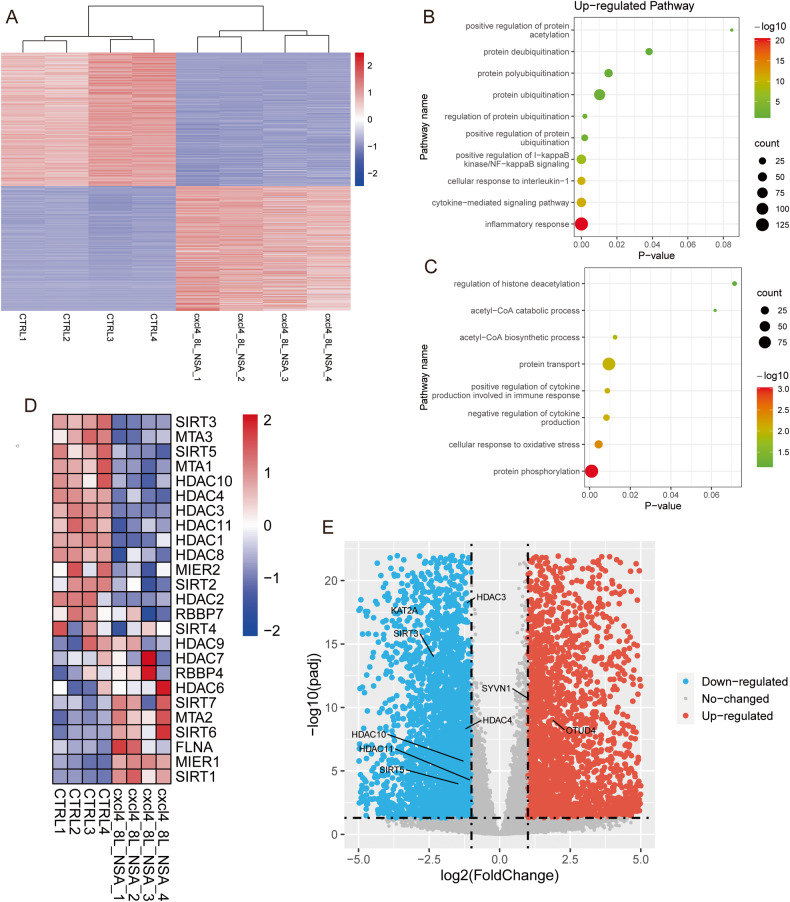
Inflammatory stimulation influences post-translational modification signaling. **A** Heatmap of all DEGs (p-values < 0.05 and |Log2 fold change| > 1) identified through the GSE201909 dataset. Red dots denote upregulated DEGs, while blue dots represent downregulated DEGs. **B**, **C** Gene ontology (GO) enrichment analysis of the DEGs in (**A**) (−lg P values). **D** Heatmap showing the alterations in the expression of specified genes in control and stimulation groups. **E** Volcano plot of genes with differential expression after stimulation. Each dot represents an individual gene. Red dots denote upregulated DEGs, while blue dots represent downregulated DEGs.

Previous studies have demonstrated the significant involvement of posttranslational modifications in the regulation of inflammation [14, 15, 20, 21]. To examine potential post-translational modifications of GSDMD in pyroptosis, we utilized the GSE201909 database, curated by Yang C et al. Our analysis focused on four control samples, where human monocytes were unstimulated, and four samples in which human monocytes were pretreated with necrosulfonamide (NSA), an inhibitor that impedes pyroptosis through directly targeting GSDMD. Subsequently, the cells were stimulated with CXCL4 and TLR8. Compared with control groups, stimulation resulted in 6779 differentially expressed genes (DEGs), including 3275 upregulated and 3504 downregulated genes, which were identified using the cutoff (|Log2 fold change| > 1 and adjusted *p* value < 0.05) (Fig. 1A and Table S1). Subsequent gene ontology (GO) analysis unveiled the extensive upregulation and downregulation of genes associated with various post-translational modification pathways, including ubiquitination and acetylation (Fig. 1B, C). Additionally, we scrutinized the expression of several representative genes linked to ubiquitination and acetylation (Fig. 1D, E), strongly suggesting that ubiquitination and acetylation play a pivotal role in pyroptosis. Considering that GSDMD functions as a pivotal downstream effector of inflammasomes, our objective was to explore the possibility of acetylation or deacetylation of GSDMD during pyroptosis, especially in light of our previous investigation into GSDMD ubiquitination, which we found to be mediated by the E3 ligase SYVN1.

**Fig. 2:**
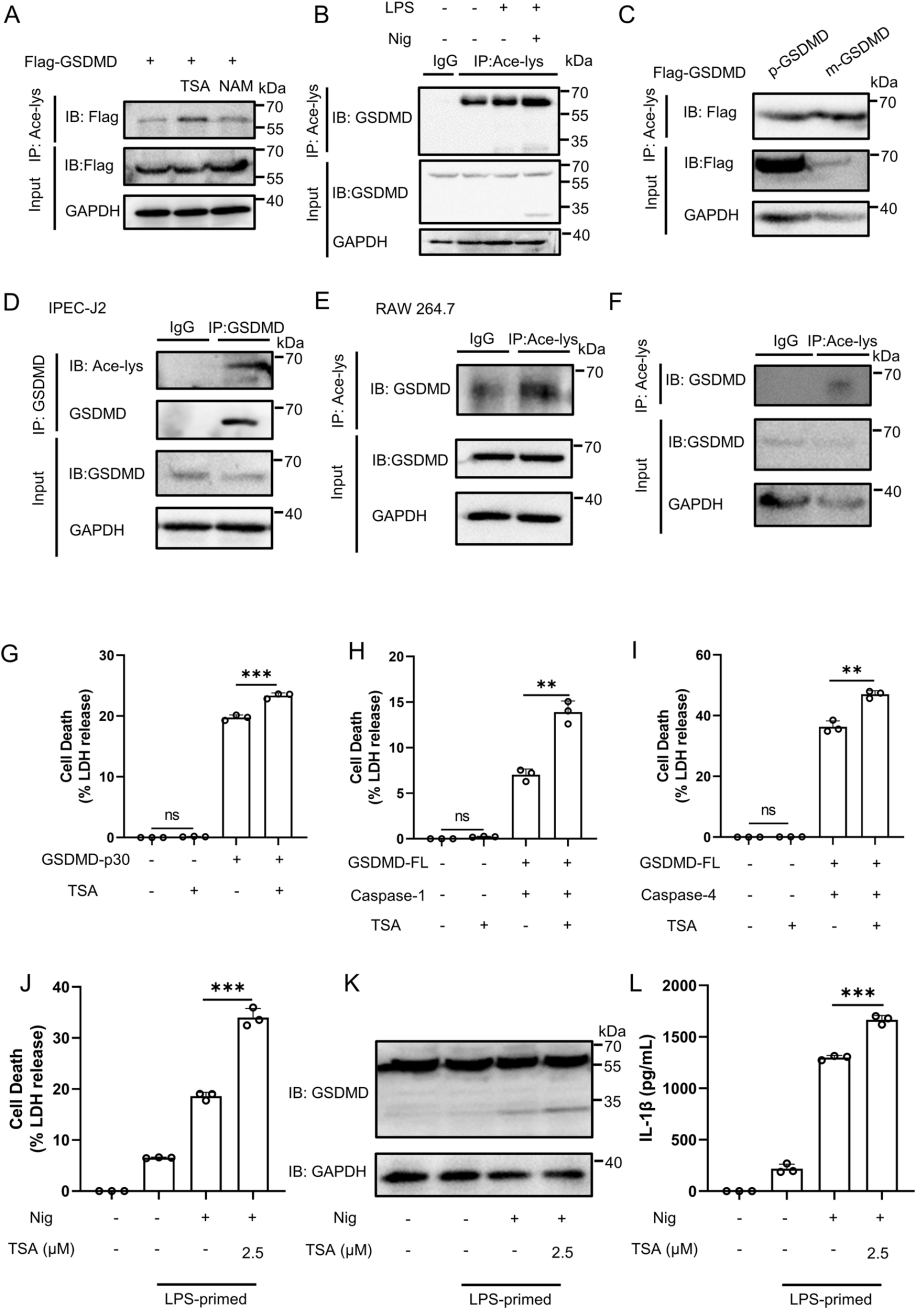
Acetylation of GSDMD affects pyroptosis. **A** Acetylation of exogenous Flag-GSDMD in HEK293T cells treated with deacetylase inhibitors TSA or NAM. Flag-GSDMD was immunoprecipitated with anti-acetyl-lys antibody (Ace-lys), and the precipitates were analyzed using an anti-flag antibody. **B** Acetylation of endogenous GSDMD in THP-1 cells. GSDMD acetylation was analyzed by immunoprecipitation with an anti-acetyl-lys antibody followed by western blotting. **C** Acetylation of exogenous Flag-GSDMD from pig or mouse in HEK293T cells. GSDMD acetylation was analyzed by immunoprecipitation with an anti-acetyl-lys antibody followed by western blotting. **D**, **E** Acetylation of endogenous GSDMD in IPEC-J2 (D), RAW264.7 (**E**) cells. GSDMD acetylation was analyzed by immunoprecipitation with an anti-acetyl-lys antibody followed by western blotting. **F** Acetylation of endogenous mouse-GSDMD from mouse lung. **G**–**I** HEK293T cells transfected with GSDMD-p30 or GSDMD-FL (full length) and Caspase-1 or GSDMD-FL and Caspase-4 were treated with TSA (10 μM). LDH release was analyzed using an LDH release assay. **J**–**L** THP-1 cells were incubated with 500 ng/mL LPS and TSA for 4 h and then another 1 h for 10 μM Nigericin. The supernatants were collected and analyzed by LDH release assay (**J**) and ELISA for IL-1β (**L**). Cell lysates were analyzed by immunoblotting (**K**). **** stands for *P* < 0.0001, *** stands for P < 0.001, ^**^ stands for P < 0.01, ^*^ stands for P < 0.05 and ns stands for no significant difference (unpaired t test). Data shown are mean ± SD from one representative experiments performed in triplicate.

We first investigated the acetylation status of GSDMD by performing an analysis on HEK293T cells. In these cells, we expressed exogenous GSDMD and then treated them with trichostatin A (TSA), a broad-spectrum inhibitor of HDAC family deacetylases, and nicotinamide (NAM), an inhibitor of SIRT family deacetylases. Through immunoprecipitation using a specific antibody against acetylated lysine, robust acetylation of GSDMD was observed in TSA-treated cells, while no significant acetylation was detected in NAM-treated cells (Fig. 2A). Additionally, in THP-1 cells treated with LPS and nigericin, which activate the canonical NLRP3 inflammasome, we observed a noticeable increase in the acetylation of endogenous GSDMD (Fig. 2B). Subsequently, we investigated the acetylation status of GSDMD in other species. HEK293T cells were separately transfected with GSDMD from pig and mouse. Following the transfections, immunoprecipitation was carried out. The results depicted in Fig. 2C demonstrated that GSDMD from both pig and mouse can undergo acetylation. Our findings confirmed the acetylation of GSDMD in pig and mouse cells, specifically IPEC-J2 cells and RAW264.7 cells, respectively (Fig. 2D, E). We also investigated the acetylation status of GSDMD in the mouse lung, and the findings provided additional support for GSDMD acetylation (Fig. 2F). Collectively, these results provide evidence that GSDMD undergoes acetylation.

As GSDMD is an executor of pyroptosis, we next evaluated the effect of GSDMD acetylation on pyroptosis. HEK293T cells were transfected with GSDMD-p30 or GSDMD-full length (GSDMD-FL), along with Caspase-1 or Caspase-4, and treated with or without TSA. Our findings demonstrated that TSA treatment significantly enhanced the release of LDH (lactate dehydrogenase) and the staining of propidium iodide (PI), indicating an increase in pyroptotic cell death (Fig. 2G–I and Supplementary Fig. 1). Similar results were obtained when HEK293T cells were transfected with p-GSDMD or m-GSDMD constructs (Supplementary Fig. 2A–D). In addition, we investigated pyroptotic cell death triggered by inflammasome activation. Initially, THP-1 cells were treated with TSA to avoid potential interference from drug toxicity during the experiment (Supplementary Fig. 3A, B). Following priming with LPS, the cells were stimulated with the NLRP3 inflammasome activator nigericin (Nig), leading to LDH release, GSDMD cleavage, and IL-1β secretion, all significantly enhanced by TSA treatment (Fig. 2J–L). Similarly, when THP-1 cells were primed with Pam3CSK4 and subsequently transfected with LPS to activate the noncanonical NLRP3 inflammasome, similar results were observed (Supplementary Fig. 2E–G). To assess the effect of TSA on other inflammasomes, we employed poly (dA:dT) as a trigger for the AIM2 inflammasome and flagellin as an NLRC4 inflammasome activator. The results demonstrated that LDH release, GSDMD cleavage, and IL-1β secretion induced by inflammasome activators in TSA-treated THP-1 cells were significantly increased (Supplementary Fig. 2H–M). Collectively, these findings suggest that GSDMD acetylation promotes pyroptosis.

### Acetylation of the K248 residue of GSDMD modulates pyroptosis

**Fig. 3:**
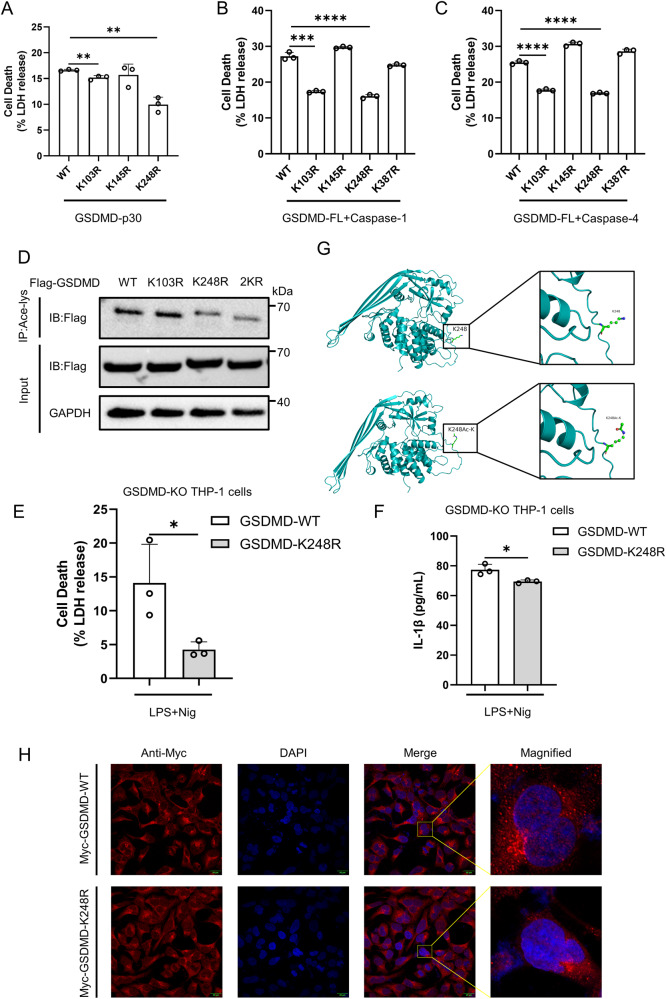
GSDMD is acetylated at Lysine 248. HEK293T cells were transfected with GSDMD-p30 mutants (**A**) or GSDMD-FL mutants and Caspase-1 (**B**) or GSDMD-FL mutants and Caspase-4 (**C**). The release of LDH in the culture medium was detected. **D** Acetylation of GSDMD mutants expressed in HEK293T cells. PMA-differentiated GSDMD-KO THP-1 macrophages were infected with Flag-GSDMD-FL-WT/K248R-Lentivirus, followed with LPS and nigericin treatment. The supernatants were collected and analyzed by LDH release assay (**E**) and ELISA for IL-1β (**F**). **G** Domain organization and ribbon diagrams of GSDMD-WT (left) and GSDMD-K248Ac-K (right). The acetylated GSDMD structure was constructed using the Structure Editing module of UCSF Chimera and optimized using Minimize Structure. **H** Immunofluorescence microscopy and nuclear staining (with the DNA-binding dye DAPI) of HEK293T cells transfected with expression plasmids for Myc-GSDMD-WT or Myc-GSDMD-K248R. Scale bars, 20 μm. **** stands for P < 0.0001, *** stands for P < 0.001, ** stands for P < 0.01, ^*^ stands for P < 0.05 (unpaired t test). Data shown are mean ± SD from one representative experiments performed in triplicate.

To identify potential acetylation sites in GSDMD, we employed mass spectrometry analysis on Flag-GSDMD obtained from TSA-treated HEK293T cells and utilized GPS-PAIL 2.0 [22], a software for predicting lysine modification sites. The results revealed four potential lysine residues: K103, K145, K248 and K387. Sequence comparison revealed that Lys103, Lys145 and Lys 248 are conserved acetylation motifs in GSDMD orthologs (Supplementary Fig. 4). Thus, we constructed several mutants (K103R, K145R, K248R and K387R) based on the GSDMD-full length or GSDMD-p30. Our results showed that the K103R and K248R mutants exhibited reduced pyroptotic activity compared to wildtype GSDMD (GSDMD-WT), whereas the K145R and K387R mutants did not show significant changes (Fig. 3A–C). Furthermore, the K248R mutant based on GSDMD-p30 exhibited a more substantial reduction in pyroptotic activity compared to the K103R mutant (Fig. 3A). Moreover, among the mutants tested, only the GSDMD-K248R mutant demonstrated a significant decrease in acetylation when compared to GSDMD-WT, unlike GSDMD-K103R or GSDMD-K103/248R mutants (Fig. 3D). This observation implies that Lysine 248 plays a pivotal role as an acetylation site in GSDMD. Additionally, reconstruction of GSDMD-K248R in GSDMD-KO THP-1 cells caused milder cell death than GSDMD-WT (Fig. 3E, F). Additionally, we developed a structural model of GSDMD with an acetyl group attached to the K248 residue (Fig. 3G). Immunofluorescence analysis demonstrated that GSDMD-K248R did not affect its localization compared to GSDMD-WT (Fig. 3H). Hence, these findings suggest that Lys 248 is one of the most critical residues for acetylation.

### Histone deacetylase 4 inhibits GSDMD-mediated pyroptosis

**Fig. 4:**
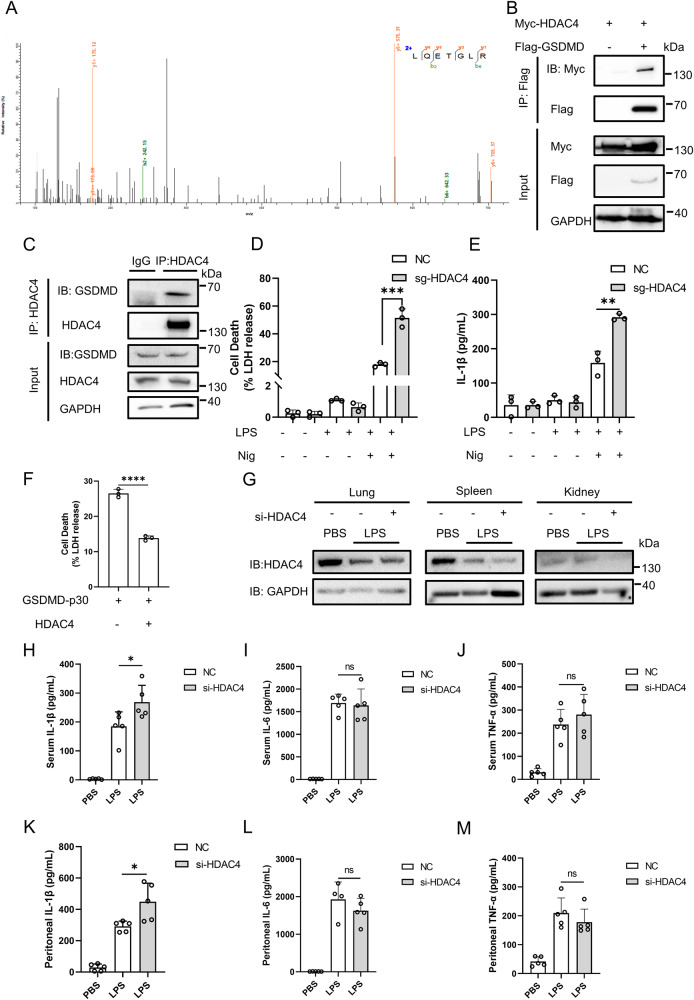
HDAC4 inhibits pyroptosis. **A** Mass spectrometry analysis of Flag-tagged GSDMD immunoprecipitated by anti-Flag antibody in HEK293T cells treated with TSA. HDAC4 was identified by marker peptide “LQETGLR”. **B** IB of total cell lysates (input) and proteins immunoprecipitated with anti-Flag resin from HEK293T cells transfected with Myc-HDAC4 and Flag-GSDMD. **C** IB of total lysates (input) and proteins immunoprecipitated with control or anti-HDAC4 antibodies in THP-1 cells. LDH release (**D**) and ELISA (**E**) of IL-1β in NC or sg-HDAC4 THP-1 cells treated with LPS and Nig. **F** LDH release of HEK293T cells transfected with GSDMD-p30, together with or without HDAC4. **G** Immunoblot analysis of the protein level of HDAC4 in various organs of control or HDAC4-knockdown mice after LPS injection for 6 h. **H**–**M** ELISA analysis of IL-1β, TNF-α and IL-6 secretion from the serum or peritoneal lavage fluid of control or HDAC4-knockdown mice after LPS injection for 6 h. (mice: n = 5 for each group). ^****^stands for P < 0.0001, ^*^ stands for P < 0.05 and ns stands for no significant difference (unpaired t test). Data shown are mean ± SD from one representative experiments performed in triplicate.

As previously mentioned, the acetylation of GSDMD markedly increased with TSA treatment rather than NAM, suggesting the involvement of HDAC family deacetylases. To identify the specific deacetylase of GSDMD, we performed a mass spectrometry analysis of Flag-tagged GSDMD with or without TSA treatment. This analysis identified histone deacetylase 4 (HDAC4) as a specific deacetylase of GSDMD by marker peptide “LQETGLR” (Fig. 4A). Co-immunoprecipitation analysis confirmed a significant interaction between GSDMD and HDAC4 in HEK293T cells (Fig. 4B). We also observed this interaction between endogenous HDAC4 and GSDMD in THP-1 cells (Fig. 4C), as well as in pigs and mice (Supplementary Fig. 5A, B). Immunofluorescence analysis showed that GSDMD and HDAC4 co-localized well in cytoplasm (Supplementary Fig. 5C). We further investigated the effect of HDAC4 on pyroptosis induced by GSDMD from different species. Our results demonstrated that HDAC4 significantly inhibited pyroptosis induced by GSDMD derived from various species (Fig. 4F and Supplementary Fig. 5D–I). HDAC4 co-transfection also reduced PI uptake (Supplementary Fig. 6). Moreover, we transfected HEK293T cells with sg-HDAC4 and tested its efficiency (Supplementary Fig. 5J). Then we infected THP-1 cells with the viral supernatant from HEK293T cells. We found that sg-HDAC4 THP-1 cells showed severer cell death after stimulation of LPS and Nig (Fig. 4D, E and Supplementary Fig. 5K). To further certify the above findings, an in vivo model was employed. First, the efficiency of HDAC4 knockdown by siRNA was verified at the protein level in NIH-3T3 cells and the results indicated a significant reduction in HDAC4 expression with si-HDAC4#1 (Supplementary Fig. 5L). Subsequently, si-HDAC4#1 or negative control siRNA was intraperitoneally injected into mice, resulting in decreased HDAC4 expression in various organs (Fig. 4G). Mice were then injected with LPS (20 mg/kg) for 6 h before serum and peritoneal lavage fluid were collected. We found that the knockdown of HDAC4 promoted the secretion of IL-1β, while the production of TNF-α and IL-6 remained unaffected (Fig. 4H–M).

To further validate the involvement of HDAC4 in the deacetylation of GSDMD and its inhibitory role in pyroptosis, we utilized LMK-235, a specific inhibitor of HDAC4. Treatment of LMK-235 could promote LDH release in HEK293T cells transfected with GSDMD-p30 or GSDMD-FL and Caspase-1 or GSDMD-FL and Caspase-4 (Supplementary Fig. 7A–C). We also treated THP-1 cells with LMK-235 to check if the drug had effect on the release of LDH and IL-1β (Supplementary Fig. 3C, D). Importantly, the effects of LMK-235 on inflammasome-mediated pyroptosis, including NLRP3, AIM2, and NLRC4, were comparable to those of TSA, further supporting the inhibitory role of HDAC4 in pyroptosis (Supplementary Fig. 7D–O). The results above suggest that HDAC4 inhibits pyroptosis both in vivo and in vitro.

### HDAC4 interacts with GSDMD in multiple domains

**Fig. 5:**
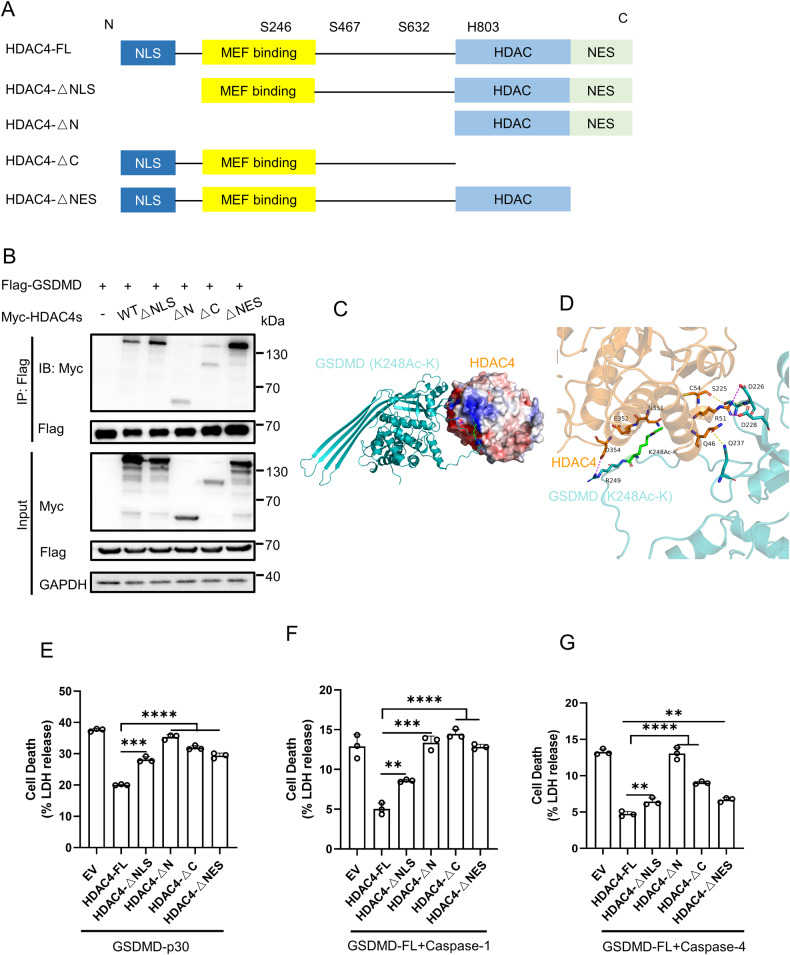
Mapping the functional domain of HDAC4 in GSDMD-mediated pyroptosis. **A** Schematic representation of HDAC4 and its mutants. **B** IB of total cell lysates (input) and proteins immunoprecipitated with anti-Flag resin from HEK293T cells transfected with Myc-HDAC4 or its mutants and Flag-GSDMD. **C**, **D** Structural model of the interaction of K248-acetylated GSDMD and HDAC4 (PDB: 2VQW). Blue represents the electropositive region, and red represents electronegative region (**C**). 3D display of interaction key amino acids (**D**), hydrogen bonds are represented by yellow dotted lines, and salt bridges are represented by magenta dotted lines. Acetylated lysine K248Ac-K was highlighted with green. The docking models were analyzed using HDOCK Server. LDH release of HEK293T cells transfected with GSDMD-p30 (**E**) or GSDMD-FL and Caspase-1 (**F**) or GSDMD-FL and Caspase-4 (**G**), together with HDAC4-WT or its mutants. **** Stands for P < 0.0001, *** stands for P < 0.001, ** stands for P < 0.01 (unpaired t test). Data shown are mean ± SD from one representative experiments performed in triplicate.

To identify the critical domain of HDAC4 responsible for its interaction with GSDMD, we generated various deletion mutants of HDAC4 (Fig. 5A) and examined their binding with GSDMD through co-immunoprecipitation experiments. Surprisingly, all mutants retained the ability to interact with GSDMD, suggesting the presence of multiple binding sites between GSDMD and HDAC4 (Fig. 5B). Structural modeling analysis further revealed the detailed interactions between GSDMD K248Ac-K and HDAC4 (Fig. 5C, D). Notably, several key interactions were identified, including a salt bridge between R249 of GSDMD and D354 on HDAC4, a salt bridge and hydrogen bond between D228 of GSDMD and R51 on HDAC4, as well as hydrogen bonds between Q237 and S225 of GSDMD and Q46 and C54 on HDAC4. Furthermore, the acetylated K248 residue of GSDMD exhibited van der Waals contact with N351 and E352 on HDAC4, facilitating substrate binding. To investigate the functional implications of these interactions, we assessed the effects of HDAC4 mutants on pyroptosis. HEK293T cells were co-transfected with GSDMD-p30 or GSDMD-FL and Caspase-1 or GSDMD-FL and Caspase-4, along with either HDAC4-FL or deletion mutants. LDH release assays demonstrated that HDAC4 effectively suppressed pyroptosis, while the deletion mutants partially rescued the inhibitory effect of HDAC4 (Fig. 5E–G), further supporting the notion that HDAC4 interacts with GSDMD at multiple sites. These findings provide insights into the intricate binding between HDAC4 and GSDMD and its functional implications in pyroptosis regulation.

### GSDMD is deacetylated by HDAC4

**Fig. 6:**
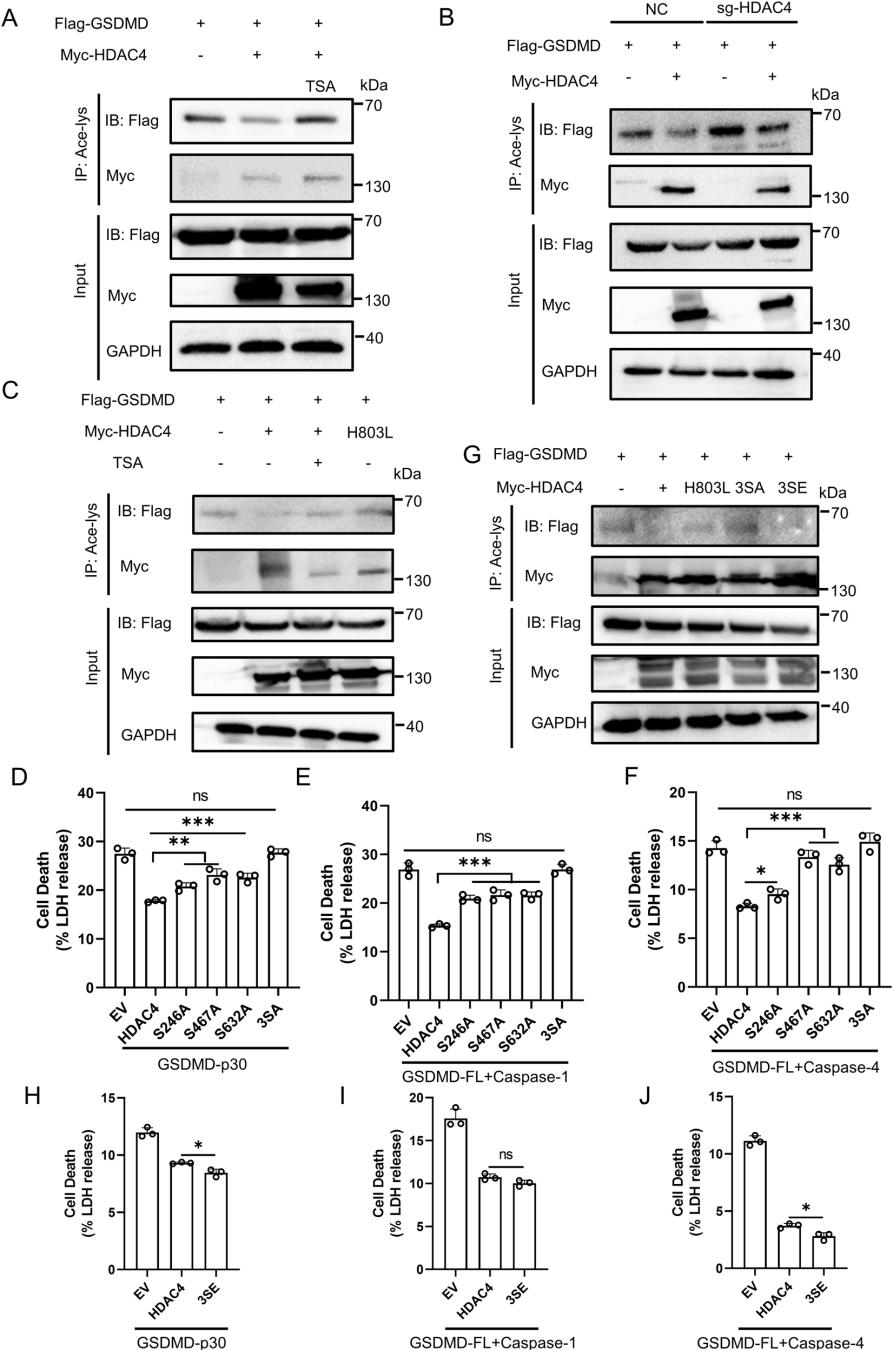
HDAC4 deacetylates GSDMD. **A** HEK293T cells were transfected with Flag-GSDMD and Myc-HDAC4, treat with or without TSA. Proteins were immunoprecipitated with anti-acetyl-lys antibody (Ace-lys), and the precipitates were analyzed using an anti-Flag or anti-Myc antibody. **B** NC and sg-HDAC4 HEK293T cells were transfected with Flag-GSDMD, together with or without Myc-HDAC4. GSDMD acetylation was analyzed by immunoprecipitation with an anti-acetyl-lys antibody followed by western blotting. **C** HEK293T cells were transfected with Flag-GSDMD and Myc-HDAC4 or HDAC4-803L, treated with or without TSA. GSDMD acetylation was analyzed by immunoprecipitation. LDH release of HEK293T cells transfected with GSDMD-p30 (**D**) or GSDMD-FL and Caspase-1 (**E**) or GSDMD-FL and Caspase-4 (**F**), together with HDAC4-WT or its mutants. **G** Acetylation of GSDMD in HEK293T cells transfected with Flag-GSDMD and Myc-HDAC4s. LDH release of HEK293T cells transfected with GSDMD-p30 (**H**) or GSDMD-FL and Caspase-1 (**I**) or GSDMD-FL and Caspase-4 (**J**), together with HDAC4-WT or HDAC4-3SE. *** Stands for P < 0.001, ** stands for P < 0.01, * stands for P < 0.05 and ns stands for no significant difference (unpaired t test). Data shown are mean ± SD from one representative experiments performed in triplicate.

We then investigated whether the inhibitory effect of HDAC4 on pyroptosis depended on its enzymatic activity. Overexpression of HDAC4 in HEK293T cells significantly reduced GSDMD acetylation, a reduction that could be completely rescued by TSA treatment (Fig. 6A). Conversely, HEK293T cells with HDAC4 knockdown (sg-HDAC4) exhibited increased GSDMD acetylation levels (Fig. 6B). Moreover, the HDAC4-H803L mutant, which lacks HDAC enzymatic activity, failed to deacetylate GSDMD (Fig. 6C). Furthermore, since HDAC4 shuttles between nucleus and cytoplasm [23], and the phosphorylation of HDAC4 at Ser 246, Ser 467 and Ser 632 is pivotal for it to export to cytoplasm [24, 25], we aimed to investigate the significance of these three sites in regulating HDAC4’s activity. We generated HDAC4 mutants with alanine substitutions at these phosphorylation sites (S246A, S467A, S632A) as well as a triple mutant (3SA). We found that these three sites all had effects on pyroptosis and the 3SA mutant almost completely lost the inhibitory capacity on pyroptosis (Fig. 6D–F), while the phosphomimetic mutant (3SE) was still capable of inhibiting pyroptosis (Fig. 6H–J). Immunofluorescence analysis showed that HDAC4 was localized in both the cytoplasm and nucleus, but co-expression with GSDMD caused a redistribution of HDAC4 to the cytoplasm (Supplementary Fig. 5C). It is possible that phosphomimetic mutation of HDAC4 abrogated its translocation from the nucleus to cytoplasm, thereby reducing the opportunity for interaction between HDAC4 and GSDMD. Furthermore, we observed that the three phosphorylation sites were also important for the deacetylation activity of HDAC4 (Fig. 6G). Thus, our data implies that Ser246, Ser467, and Ser632 residues of HDAC4 are functionally crucial for its ability to deacetylate GSDMD and inhibit pyroptosis.

### Protein phosphatase 1 regulates phosphorylation of HDAC4

**Fig. 7:**
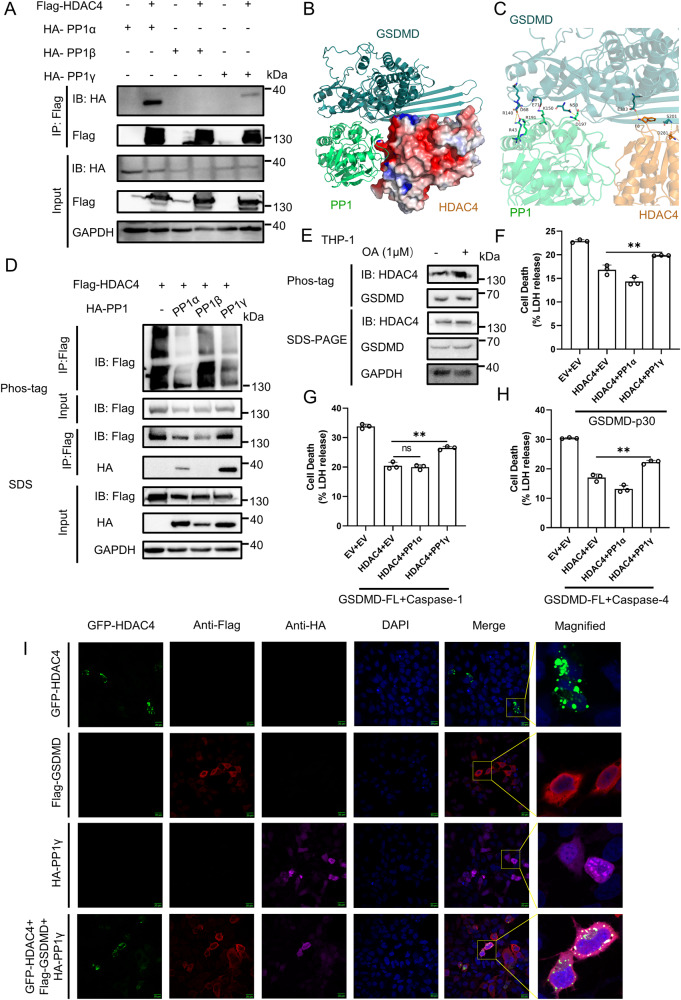
PP1 dephosphorylates HDAC4. **A** Interaction of HDAC4 and PP1. IB of total cell lysates (input) and proteins immunoprecipitated with anti-Flag resin from HEK293T cells transfected with Flag-HDAC4 and HA-PP1. **B**, **C** Structural model of the complex of GSDMD, HDAC4 and PP1 (PDB: 4MOV). The docking models were analyzed using HDOCK Server. **D** Analysis of phosphorylation of HDAC4 in HEK293T cells transfected with Flag-GSDMD and HA-PP1. The cells were lysed, and their proteins were separated by Phos-tag PAGE and subjected to Western blot analysis. **E** THP-1 cells were treated with or without Okadaic acid (OA) and then lysed. Proteins were separated by Phos-tag PAGE and subjected to Western blot analysis. HEK293T cells were first transfected with HDAC4 and PP1. After 24 h, cells were then transfected with GSDMD-p30 (**F**) or GSDMD-FL and Caspase-1 (**G**) or GSDMD-FL and Caspase-4 (**H**). The supernatants were collected for LDH assay. **I** Immunofluorescence microscopy and nuclear staining (with the DNA-binding dye DAPI) of HEK293T cells transfected with expression plasmids for GFP-HDAC4, Flag-GSDMD and HA-PP1γ. Scale bars, 20 μm. ** stands for P < 0.01 and ns stands for no significant difference (unpaired t test). Data shown are mean ± SD from one representative experiments performed in triplicate.

As the phosphorylation of HDAC4 was relevant to its ability of deacetylation, we sought to find the phosphatase involved in the process. It is known that Protein phosphatase 1 (PP1) is responsible for the regulation of GSDMD phosphorylation and inhibits pyroptosis [26]. Given that HDAC4 also inhibits pyroptosis, we hypothesize that PP1 interacts with HDAC4. We first confirmed that PP1α and PP1γ interacted with GSDMD (Supplementary Fig. 8A). We then examined the interaction between PP1 and HDAC4 and found that HDAC4 indeed interacted with both PP1α and PP1γ, similar to its interaction with GSDMD (Fig. 7A). Immunofluorescence analysis also showed that PP1 and HDAC4 co-localized well in cells (Supplementary Fig. 8B). Structural modeling analysis revealed that they exhibited good binding, primarily through hydrogen bonds and van der Waals contacts (Supplementary Fig. 8C–E). We further explored the trilateral interaction of HDAC4, GSDMD and PP1. Immunofluorescence analysis demonstrated their co-localization in the cytoplasm (Fig. 7I). Through structural modeling, we observed that GSDMD and HDAC4 formed two hydrogen bonds, while two salt bridges and three hydrogen bonds were formed between HDAC4 and PP1 (Fig. 7B, C). To investigate the phosphorylation status of HDAC4 in the presence or absence of PP1, we performed Phos-tag SDS-PAGE, which allows detection of phosphorylation levels based on the mobility shift of phosphorylated proteins. HDAC4 co-transfection with PP1α and PP1γ showed less mobility shift (Fig. 7D), suggesting that HDAC4 is dephosphorylated by PP1α and PP1γ. We conducted an experiment to investigate whether PP1 dephosphorylates HDAC4 by treating THP-1 cells with okadaic acid (OA), a known PP1 inhibitor. We separated the proteins using Phos-tag PAGE and subsequently conducted Western blot analysis. The data in Fig. 7E clearly demonstrate that treatment with OA led to an increase in the phosphorylation of HDAC4. To assess the effect of PP1 on HDAC4-mediated pyroptosis inhibition, HEK293T cells were first co-transfected with HDAC4 and PP1α or PP1γ for 24 h, followed by transfection of GSDMD-p30 or GSDMD-FL and Caspase-1 or GSDMD-FL and Caspase-4. The results showed that only PP1γ counteracted the inhibitory effect of HDAC4 on pyroptosis (Fig. 7F–H). Moreover, treatment with OA further promoted the inhibition of HDAC4 on pyroptosis (Supplementary Fig. 8F–H). Taken together, the above results indicate that PP1 participates in the processing of GSDMD deacetylation by dephosphorylating HDAC4.

### Acetylation of GSDMD facilitates the ubiquitination of GSDMD

**Fig. 8:**
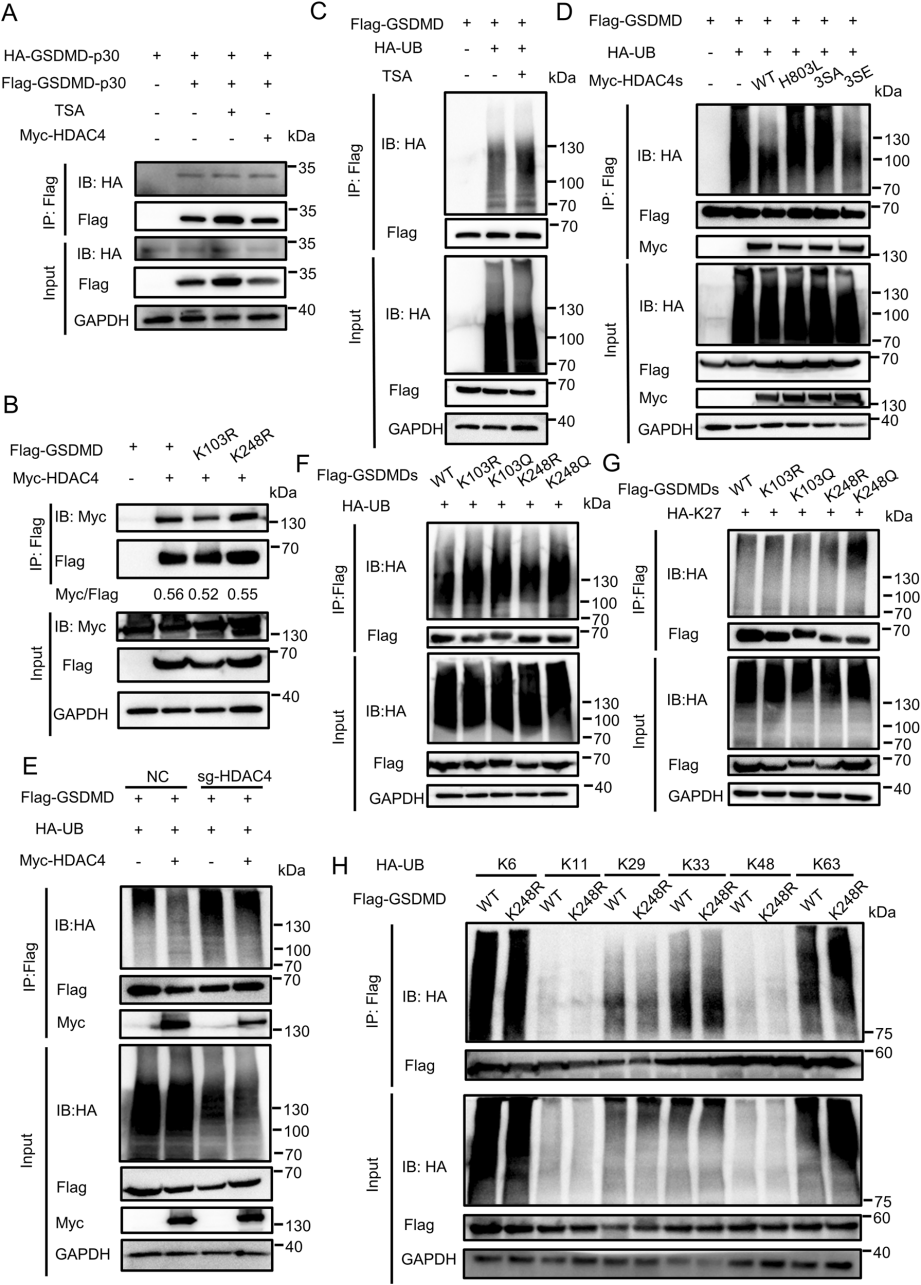
Acetylation of GSDMD promotes its ubiquitination. **A** IB of total cell lysates (input) and proteins immunoprecipitated with anti-Flag resin from HEK293T cells transfected with Flag-GSDMD-p30 and HA-GSDMD-p30, together with or without Myc-HDAC4 or TSA. **B** IB of total cell lysates (input) and proteins immunoprecipitated with anti-Flag resin from HEK293T cells transfected with Myc-HDAC4 and Flag-GSDMD or its mutants. **C** IB of total cell lysates (input) and proteins immunoprecipitated with anti-Flag resin from HEK293T cells transfected with Flag-GSDMD and HA-UB, treat with or without TSA. **D** IB of total cell lysates (input) and proteins immunoprecipitated with anti-Flag resin from HEK293T cells transfected with Flag-GSDMD and HA-UB, together with HDAC4 or its mutants. **E** NC and sg-HDAC4 HEK293T cells were transfected with Flag-GSDMD, together with or without Myc-HDAC4. GSDMD ubiquitination was analyzed by immunoprecipitation with an anti-GSDMD antibody followed by western blotting. IB of total cell lysates (input) and proteins immunoprecipitated with anti-Flag resin from HEK293T cells transfected with Flag-GSDMD and HA-UB (**F**) or HA-K27 (**G**). All results shown are representative of at least three independent experiments. **H** IB of total cell lysates (input) and proteins immunoprecipitated with anti-Flag resin from HEK293T cells transfected with Flag-GSDMD or Flag-GSDMD-K248R and HA-K6/ K11/ K29/ K33/ K48/ K63.

Protein acetylation and ubiquitination are often associated. Cross-talk between lysine acetylation and ubiquitination have been proved to be an important regulatory mechanism in regulating protein functions [15, 18, 19, 27–29]. We first checked whether the acetylation of GSDMD-p30 affected its oligomerization. The results showed that HDAC4 transfection or TSA-treatment did not affect GSDMD-p30 oligomerization (Fig. 8A). Furthermore, we confirmed that HDAC4 did not impact the degradation of GSDMD (Supplementary Fig. 9A). We also detected the binding between GSDMD-WT or GSDMD mutants and HDAC4, and the results indicated that the binding was not affected (Fig. 8B). Subsequently, we explored the relationship between the acetylation and ubiquitination of GSDMD. TSA-treatment promoted the ubiquitination of GSDMD (Fig. 8C). Moreover, HDAC4 was able to inhibit GSDMD ubiquitination, while HDAC-H803L and HDAC4-3SA mutants were not able to exert this inhibitory effect (Fig. 8D). The ubiquitination level of GSDMD in HEK293T cells transfected with sg-HDAC4 was lower than cells transfected with NC (Fig. 8E). Then we constructed the acetylation-mimetic mutants GSDMD-K103Q and GSDMD-K248Q and examined their ubiquitination level in HEK293T cells. Compared with GSDMD-WT, GSDMD-K248Q displayed a higher extent of ubiquitylation (Fig. 8F). As we have proved that GSDMD could be ubiquitinated with K27-linked polyubiquitin chains [16], we next investigated whether the acetylation of GSDMD affected its K27-linked ubiquitination. We observed that GSDMD-K248Q similarly displayed an elevated level of K27-linked ubiquitination (Fig. 8G). Conversely, no significant differences were detected in other types of ubiquitination upon transfection with either GSDMD-WT or GSDMD-K248R (Fig. 8H). These findings suggest that acetylation of GSDMD promotes its ubiquitination, which, in turn, facilitates GSDMD-mediated pyroptosis. This indicates the presence of a regulatory cross-talk between acetylation and ubiquitination in modulating the function of GSDMD.

## Discussion

GSDMD acts downstream of inflammasome activation and is responsible for forming transmembrane pores and cytokine secretion. However, the regulation of GSDMD remains elusive. We have previously demonstrated that GSDMD is modified by ubiquitination, thereby promoting pyroptosis [16]. Occasionally, we have also observed the involvement of acetyltransferases and deacetylases in GSDMD ubiquitination. Thus, we reveal a previously unknown post-translational modification of GSDMD. GSDMD can be acetylated in the resting state and the acetylation level of GSDMD increases after NLRP3 inflammasome activation. Subsequently, acetylation of GSDMD promotes pyroptotic cell death.

Acetylation was initially described as an epigenetic mechanism modulating nucleosomal DNA accessibility and transcription. Subsequently, it was revealed that acetylation plays an essential role in regulating physiological processes, including the cell cycle, DNA damage repair, and cellular signaling [30–33]. Acetylation of proteins has effects on protein–protein interactions and protein degradation. Both proteasome-dependent and proteasome-independent protein degradation can be affected by acetylation. PEPCK1 acetylation results in PEPCK1 ubiquitination and degradation, while acetyltransferases CBP, p300 and TIP60 and deacetylases HDAC6 and SIRT1 are important regulators of autophagy [34, 35]. In this study, we found that GSDMD was acetylated at Lys 248. HDAC4 was also identified as the deacetylase of GSDMD. HDAC4 directly interacts with GSDMD at multiple sites and deacetylates it. Intriguingly, we found that the S246, S467 and S632 residues of HDAC4 played important roles in the process of deacetylation and suppressing pyroptosis. We hypothesized that these three sites affect the export of HDAC4 to the cytoplasm, where it binds to GSDMD and exerts its function. HDAC4 is reported to undergo phosphorylation by the signaling kinase TBK1/IKKε, which in turn prevents IRF3 phosphorylation [25]. However, the phosphatase that mediates the dephosphorylation of HDAC4 remains unclear at present. PP1 is found to dephosphorylate GSDMD [26]. Interestingly, we observed that PP1 binds to both HDAC4 and GSDMD, leading to the dephosphorylation of HDAC4. Therefore, PP1 not only inhibits pyroptosis by dephosphorylating GSDMD but also affects the deacetylase activity of HDAC4 on GSDMD by inhibiting HDAC4 phosphorylation, thereby preventing the translocation of HDAC4 to the cytoplasm. Furthermore, despite PP1α‘s capacity to dephosphorylate HDAC4 and induce the cytoplasmic translocation of HDAC4, it was unable to mitigate the inhibitory impact of HDAC4 on pyroptosis. This leads us to speculate that additional phosphorylation sites may exist in HDAC4 that are subject to dephosphorylation by PP1α.

Proteins undergo various PTMs, which can reciprocally affect one another. Lysine residues can undergo diverse modifications, such as acetylation, methylation, and ubiquitination. This can result in cross-talk among PTMs, where different PTMs compete for the same residue. For example, acetylation of p53 competes with ubiquitination mediated by MDM2 on the same lysine residues, thereby inhibiting its degradation through ubiquitin-proteasome pathway [36]. However, PTMs do not always behave competitively. Acetylation at K420 and K435 sites enhances p62 binding to ubiquitin and K435 acetylation also directly increases the UBA-ubiquitin affinity [19]. Our results indicated that the acetylation of GSDMD at K248 site promotes GSDMD K27-linked ubiquitination, thereby illustrating a novel example of cross-talk between acetylation and ubiquitination.

In summary, we have unraveled a previously unknown post-translational modification of GSDMD and elucidated a novel interplay between GSDMD, HDAC4, and PP1 in the regulation of pyroptosis (Supplementary Fig. 10). The post-translational modifications of GSDMD and HDAC4, including ubiquitination, acetylation, and phosphorylation, play crucial roles in modulating pyroptosis. The interplay between different PTMs and the involvement of HDAC4 and PP1 add complexity to the regulation of pyroptosis and further our understanding of this important biological process. These findings offer insights into the mechanisms underlying pyroptosis and provide opportunities to intervene in inflammatory-related diseases.

## Materials and methods

### Reagents and antibodies

Anti-HA (3724), anti-GSDMD (39754) and anti-Acetylated Lysine (9441) antibodies were purchased from Cell Signaling Technology. Anti-GAPDH antibody, HRP-labeled goat anti-mouse IgG and goat anti-rabbit IgG were from Hangzhou Fudebio. Anti-Flag M2 (F1804), anti-Flag M2 Magnetic Beads (10004D), anti-Myc (C3956) antibodies and LPS (O111:B4, L2630) were purchased from Sigma-Aldrich. Anti-FLAG antibody (rabbit source), Goat pAb to MS IgG (Chromeo 555, ab60316), Dnk pAb to Rb IgG (Alexa Fluor 647, ab150075) fluorescent secondary antibodies were obtained from Abcam. Nigericin, Pam3CSK4 (TLRL-PMS), poly(dA:dT) (TLRL-PATN) and flagellin (TLRL-EPSTFLA) were purchased from InvivoGen. ELISA kits for analysis of human IL-1β, mouse IL-1β, mouse IL-6 and mouse TNF-α were purchased from MultiSciences. CytoTox 96 LDH-release assay kit (G1780) was from Promega. Efficient eukaryotic transfection reagent VigoFect was purchased from Vigorous Biotechnology (Beijing), Lipofectamine 2000 transfection reagent was obtained from Invitrogen while Lipo8000 transfection reagent was obtained from Beyotime Biotechnology. Trichostain A (HY-15144), Nicotinamide (HY-B0150), LMK-235(HY-18998) and okadaic acid (HY-N6785) were purchased from MCE.

### Plasmid construction and transfection

Flag-GSDMD, Flag-GSDMD-p30, HA-GSDMD-p30, HA-Caspase-1, Myc-Caspase-4 and HA-tagged ubiquitin was constructed previously. Myc-HDAC4 was made by cloning human HDAC4 protein ORF (GeneBank: NM_001378415.1) into a pCMV-Myc vector using NotI and SalI restriction sites. HDAC4 mutants were constructed based on the eukaryotic expression plasmids Myc-HDAC4. The primers used in this study are shown in Supplementary Table 1. According to the manufacturer’s instructions, indicated plasmids were transfected using VigoFect, Lipofectamine 2000 or Lipo8000 separately.

### Cell culture and stimulation

Human embryonic kidney (HEK) 293T and THP-1 cells were maintained in the laboratory.All cells were cultured at 37 °C in 95% air and 5% CO_2_. HEK293T cells, IPEC-J2 cells, RAW264.7 cells and NIH-3T3 cells were cultured in Dulbecco’s modified Eagle’s medium (DMEM) with 10% FBS (Excell) and 1% penicillin–streptomycin (Hyclone). Human monocyte cell line THP-1 cells were cultured in RPMI 1640 medium with added 10% FBS (Gibco) and 1% penicillin-streptomycin (Hyclone). To induce inflammasome activation, 5 × 10^5^ THP-1 cells were plated in a 24-well plate overnight with 1 μM PMA, and then the medium was changed next morning. In order to activate the canonical NLRP3 inflammasome, cells were primed for 4 h with 500 ng/mL LPS and then stimulated for 1 h using 10 μM Nigericin. For activation of the non-canonical inflammasome, cells were primed for 4 h with 250 ng/mL Pam3CSK4, after which the medium was replaced and cells were transfected with 2 μg/mL LPS for 6 h. For activation of AIM2 inflammasome and NLRC4 inflammasome, cells were primed for 4 h with 500 ng/mL LPS, followed by stimulating for 6 h with 2 μg/mL poly(dA:dT) or flagellin.

### Immunoblotting

Cells were harvested and lysed in RIPA lysis buffer (Beyotime Biotechnology) added with 1% PMSF (Beyotime Biotechnology). Proteins were separated on the 10% SDS-PAGE gel (Hangzhou Fudebio) and transferred onto the PVDF membranes (Bio-rad). Membranes were blocked in the blocking buffer (Beyotime Biotechnology), followed by staining with primary antibodies and positioning with secondary antibodies. Chemiluminescent signals were captured by an ECL chemiluminescence imaging analysis system (Clinx Science Instruments).

### Co-immunoprecipitation

For GSDMD acetylation analysis, cells were lysed in IP lysis buffer (Beyotime Biotechnology) added with PMSF. Then the cell lysate was mixed with antibodies at 4 °C overnight followed by the addition of protein G beads (10003D, Invitrogen).

Otherwise, cells were lysed in IP lysis buffer added with PMSF. The supernatants were then incubated with anti-Flag binding beads (Sigma, M8823) at 4 °C. The immunocomplexes were washed and then subjected to immunoblotting analysis.

### Confocal immunofluorescence assay

HEK293T cells were seeded on coverslips in 24-well plates. After being transfected for 24 h, cells were fixed with Immunol Staining Fix Solution (Beyotime). Cells were incubated with primary antibodies overnight at 4 °C after being permeabilized and blocked. Alex Fluor 555/647-conjugated secondary antibody was incubated for 1 h. Nuclei were stained with DAPI. Confocal micrographs were imaged using a laser confocal microscope (Olympus).

### LDH assay

The supernatants of cells were collected and then applied to cytotoxicity test using CytoTox 96^®^ Reagent (Promega) according to the manufacturer’s manual. OD values were collected at 492 nm on an enzyme marker (Thermo Scientific).

### ELISA

Supernatants from stimulated cells, mice serum and peritoneal lavage fluid were applied to the detection of IL-1β/TNF-α/IL-6 according to the manufacturer’s instructions. Each trial group was conducted independently for three times.

### Phos-tag SDS-PAGE

Supplements of 50 μM phos-tag and 10 mM MnCl_2_ were added during preparation of SDS-PAGE gel. Others were same as above protocol of immunoblotting.

### Construction of HDAC4 knockout cells

HDAC4 knockout HEK293T cells were generated using the CRISPR/Cas9 technique. Vectors expressing gRNA targeting human HDAC4 were transfected into HEK293T cells. For HDAC4 knockout THP-1 cells, gRNA plasmid was co-transfected with the lentiviral packaging vectors pMD2G and PSPAX2, then introduced into HEK293T cells to produce lentivirus. After 48 h, the viral supernatants were collected and added to THP-1 cells in 6-well plates with 1 mL medium containing 8 μg/mL polybrene. The infected cells were spun at 300 × *g* for 1 h, and then 1 mL fresh media was added. After infection for 2 days, stably transfected cells were selected with GFP by flow-cytometric analysis. Single-cell sorting of transfected cells was performed using flow cytometry (MOFLO XDP).

### RNA interference

SiRNAs (Genepharma) specific for mouse HDAC4 were transfected into NIH-3T3 cells using the GP-transfect-Mate according to the manufacturer’s instructions. Then the si-HDAC4#1 (5′-3′ CACCAUCCUUACCCAACAUTT) were injected into mice (50 μg /mouse) in the presence of in vivo RNA transfection reagent (Entranster-in vivo; Engreen). siRNA sequences are shown in Supplementary Table 2.

### h-GSDMD reconstruction lentivirus

The lentivirus was produced by transfection of the pLVX-IRES-Puro-h-GSDMD-WT/h-GSDMD-K248R or control vector into HEK293T cells using Lipo8000 with pMD2G and PSPAX2. Supernatants were collected after 72 h incubation for further lentiviral concentration.

### Data preprocessing and DEGs screening

HISAT2 was used to align reads to the reference genome (GRCh37) [37]. Samtools was applied to convert sequence alignment/map (.SAM) format files into binary alignments/maps (.BAM) format [38], and featureCounts was used for quantifying gene expression [39]. Only uniquely mapped reads were used for expression quantification. According to the workflow, DEGs were screened using the “DESeq2” package [40] in R software (version 4.1.2) with the cutoff |Log2 fold change| > 1 and adjusted p value < 0.05.

### LC–MS/MS analysis

Flag-tagged GSDMD immunoprecipitants prepared from whole-cell lysates or gel-filtrated fractions were resolved on SDS-PAGE gels, and protein bands were excised. The samples were digested with trypsin, and then subject to LC-MS/MS analysis. Swissprot_Human mass spectra were used as the standard reference. Trypsin/P was used for cleavage. MS data were captured and analyzed by Shanghai Bioprofile.

### Propidium iodide staining

Cells were incubated with Propidium iodide (PI) (BD Bioscience) for 15 min under light-proof conditions after transfection with indicated plasmids for 24 h. Dyeing condition was observed under fluorescence microscope (Nikon).

### Statistical analysis

All experiments were performed independently at least three times. Data were presented as the mean ± standard deviation (SD), analyzed and used for statistical graphing by GraphPad Prism 8, the significance of differences was determined by Student’s *t*-test. The significance of differences ranked as: ***stands for *P* < 0.001, ** stands for P < 0.01, * stands for P < 0.05 and ns stands for non-significant difference.

### Reporting summary

Further information on research design is available in the Nature Research Reporting Summary linked to this article.

### Supplementary information

Supplementary Figures

Supplementary Tables

Reporting summary

Original western blots

## Data Availability

All data needed to evaluate the conclusions in the paper are present in the paper and/or the Supplementary Materials. Additional data related to this paper may be requested from the corresponding author.
