# Supplementary material for: Crosstalk of HDAC4, PP1, and GSDMD in controlling pyroptosis
Source: Cell Death Dis. 2024 Feb 7;15(2):115. doi: 10.1038/s41419-024-06505-z (PMC10850491; doi:10.1038/s41419-024-06505-z)
Supplement: Supplementary file 1 — Supplementary Figures [file 41419_2024_6505_MOESM1_ESM.pdf]

Crosstalk of HDAC4, PP1, and GSDMD in Controlling Pyroptosis

Weilv *et al*.

Supplementary Information

Inventory of Supporting Information

1. Supplementary Figures and figure legends

Supplementary Fig. 1 PI staining for Fig. 2G-I.

Supplementary Fig. 2 TSA-treatment promotes pyroptosis.

Supplementary Fig. 3 TSA and LMK-235 have no effect on cytokine secretion.

Supplementary Fig. 4 Candidate acetylation sites of GSDMD.

Supplementary Fig. 5 HDAC4 suppresses pyroptotic cell death.

Supplementary Fig. 6 PI staining for Fig. 4F.

Supplementary Fig. 7 LMK-235-treatment promotes pyroptosis.

Supplementary Fig. 8 PP1 mediated dephosphorylation of HDAC4.

Supplementary Fig. 9 HDAC4 does not affect GSDMD degradation.

Supplementary Fig. 10 Diagram of HDAC4 regulates GSDMD-mediated pyroptosis.

2. Supplementary Tables

Supplementary Table 1. DEGs list.

Supplementary Table 2. Primers used in this study for the construction of plasmids.

Supplementary Table 3. siRNA sequences for the mouse HDAC4 oligonucleotide.

1. Supplementary Figures

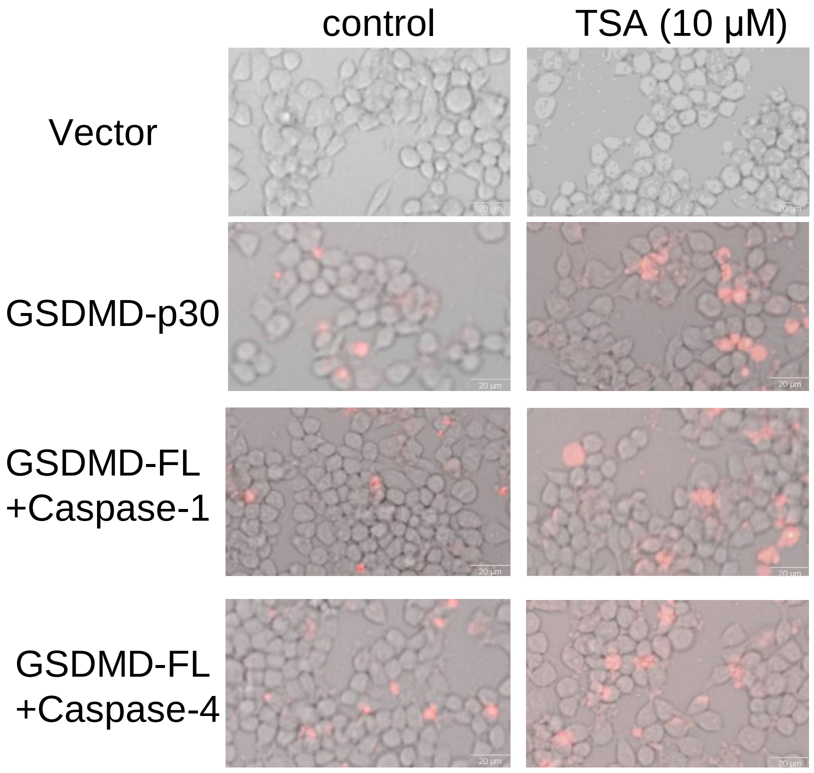

Supplementary Fig. 1 PI staining for Fig. 1G-I. PI staining of HEK293T cells after 24 h transfection with Vector or GSDMD-p30 or GSDMD-FL and Caspase-1 or GSDMD-FL and Caspase-4, treated with or without 10 μM TSA. Scale bars, 20 μm.

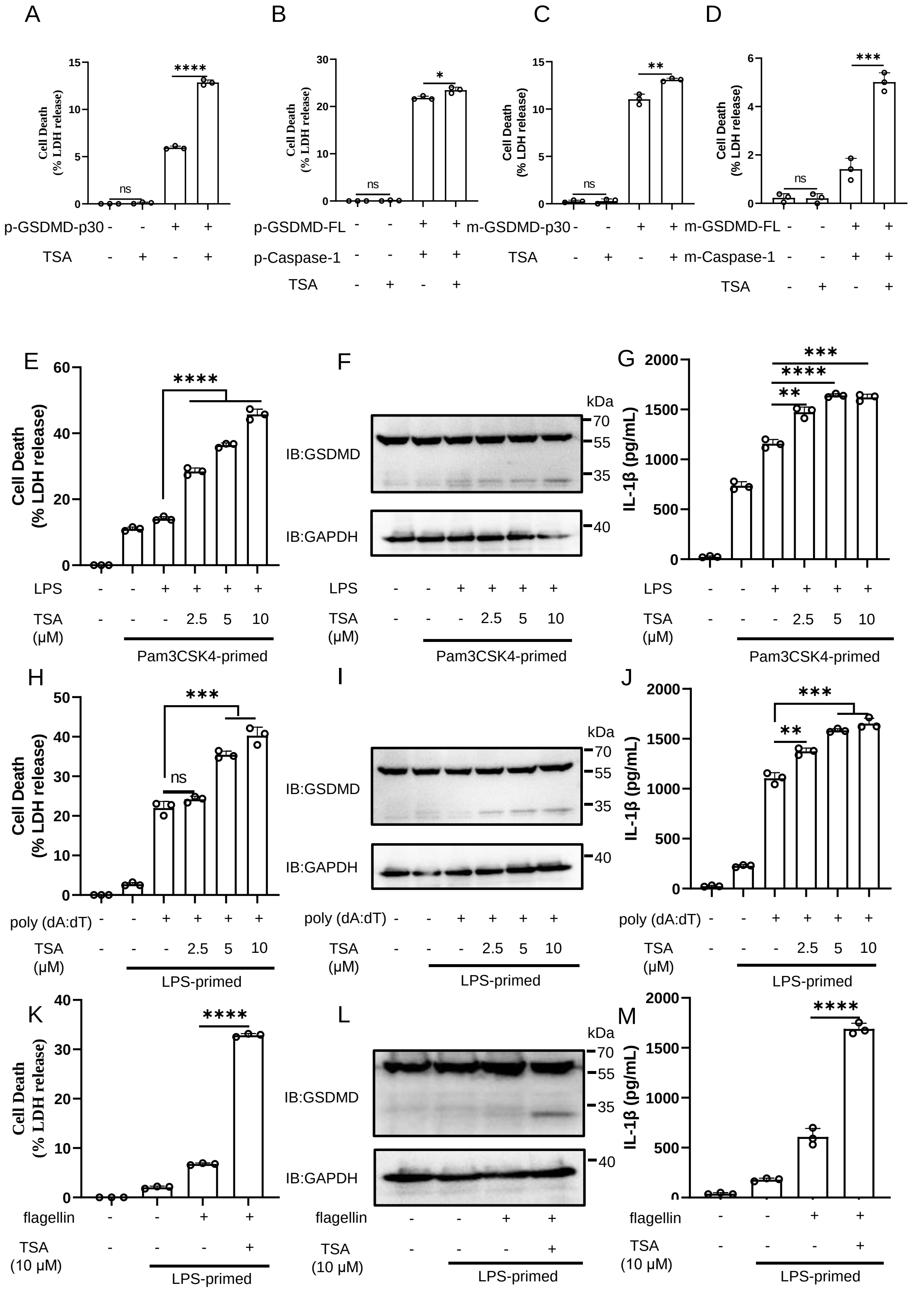

Supplementary Fig. 2 TSA-treatment promotes pyroptosis. (A-D) HEK293T cells were transfected with pig (A, B) or mouse (C, D) GSDMD-p30 or GSDMD-FL and Caspase-1, treat with or without 10 μM TSA. The supernatants were collected for LDH

assay. **(E-G)** PMA-differentiated THP-1 cells were primed with 250 ng/mL Pam3CSK4 and TSA for 4 h, and then transfected with 2 μg/mL LPS for 6 h. The supernatants were collected and analyzed by LDH release assay (E) and ELISA for IL-1β (G). Cell lysates were analyzed by immunoblotting (F). **(H-J)** PMA-differentiated THP-1 cells were primed with 500 ng/mL LPS and TSA for 4 h, and then transfected with 2 μg/mL poly(dA:dT) for 6 h. The supernatants were collected and analyzed by LDH release assay (H) and ELISA for IL-1β (J). Cell lysates were analyzed by immunoblotting (I). **(K-M)** PMA-differentiated THP-1 cells were primed with 500 ng/mL LPS and TSA for 4 h, and then transfected with 2 μg/mL flagellin for 6 h. The supernatants were collected and analyzed by LDH release assay (K) and ELISA for IL-1β (M). Cell lysates were analyzed by immunoblotting (I). ****stands for $P<0.0001$, ***stands for $P<0.001$, ** stands for $P<0.01$, * stands for $P<0.05$ and ns stands for no significant difference (unpaired t test). Data shown are mean ± SD from one representative experiments performed in triplicate.

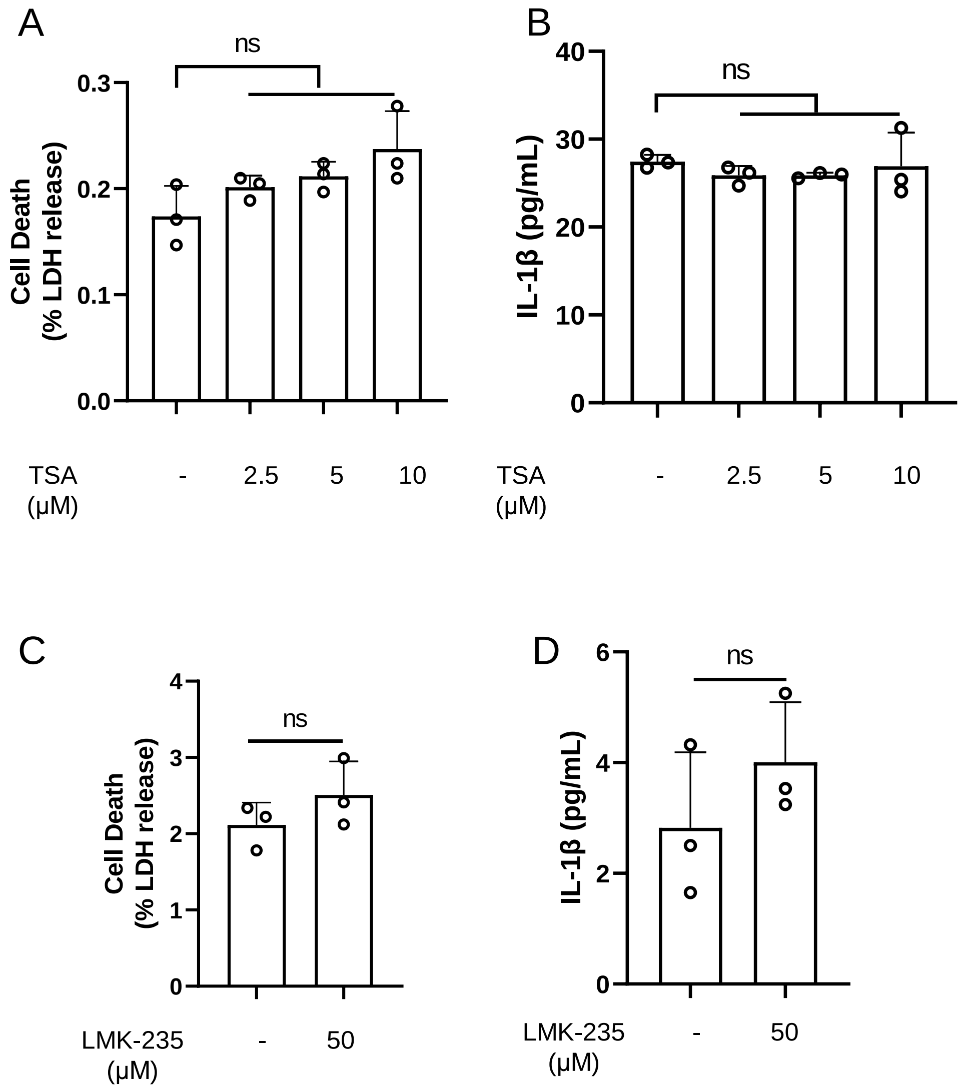

Supplementary Fig. 3 TSA and LMK-235 have no effect on cytokine secretion. (A-B) THP-1 cells were treated with TSA for 6 h, and supernatants were collected and analyzed by LDH release assay (A) and ELISA for IL-1β (B). **(C-D)** THP-1 cells were treated with LMK-235 for 6 h, and supernatants were collected and analyzed by LDH release assay (C) and ELISA for IL-1β (D). Ns stands for no significant difference (unpaired t test). Data shown are mean ± SD from one representative experiments performed in triplicate.

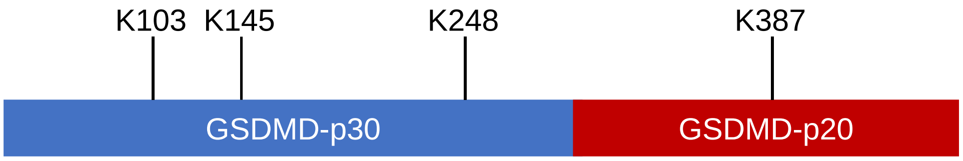

MGSAFERVVRRVVQELDHGGEFIPVTSLQSSTGFQPYCLVVRKPSSSWFW
KPRYKCVNLSIKDILEPDAAEPDVQRGRSFHFYDAMDGQIQGSVELAAPGQ
AKIAGGAAVSDSSSTSMNVYSLSVDPNTWQTLLHERHLRQPEHKVLQQLRS
RGDNVYVVTEVLQTQKEVEVTRTHKREGSGRFSLPGATCLQGEGQGHLSQ
KKTVTIPSGSTLAFRVAQLVIDSDLDVLLFPDKKQRTFQPPATGHKRSTSEGA
WPQLPSGLSMMRCLHNFLTDGVPAEGAFTEDFQGLRAEVETISKELELLDR
ELCQLLLEGLEGVLRDQLALRALEEALEQGQSLGPVEPLDGPAGAVLECLVL
SSGMLVPELAIPVVYLLGALTMLSETQHKLLAEALESQTLLGPLELVGSLLEQ
SAPWQERSTMSLPPGLLGNSWGEGAPAWVLLDECGLELGEDTPHVCWEP
QAQGRMCALYASLALLSGLSQEPH

	103	145	248	387
Human	99 GQAKIAGG	142 PEHKVLQ	245 GHKRST	384 TQHKLLA
Mouse	100 GEGKISGG	143 PENKILQ	246 DRKAVG	387 TQQQLLA
Rattus	100 GQGKISGG	143 PENKILQ	246 GHKGAV	387 TQQQLLA
Pig	99 GQGKFSGK	142 PEHKVLQ	245 GHSASH	388 TQHVLLA
Cat	99 GQGKFVSG	142 PEHKILQ	245 GHKPTS	387 TQRVLLA
Macaca	....	25 PEHKILQ	128 GHKPSR	267 TQHKLLA

Supplementary Fig. 4 Candidate acetylation sites of GSDMD. Scheme of human GSDMD protein marked with four candidate acetylation sites, including K103, K145, K248 and K387. Sequence alignment of the K103, K145, K248 and K387 sites within GSDMD orthologues of different species (below).

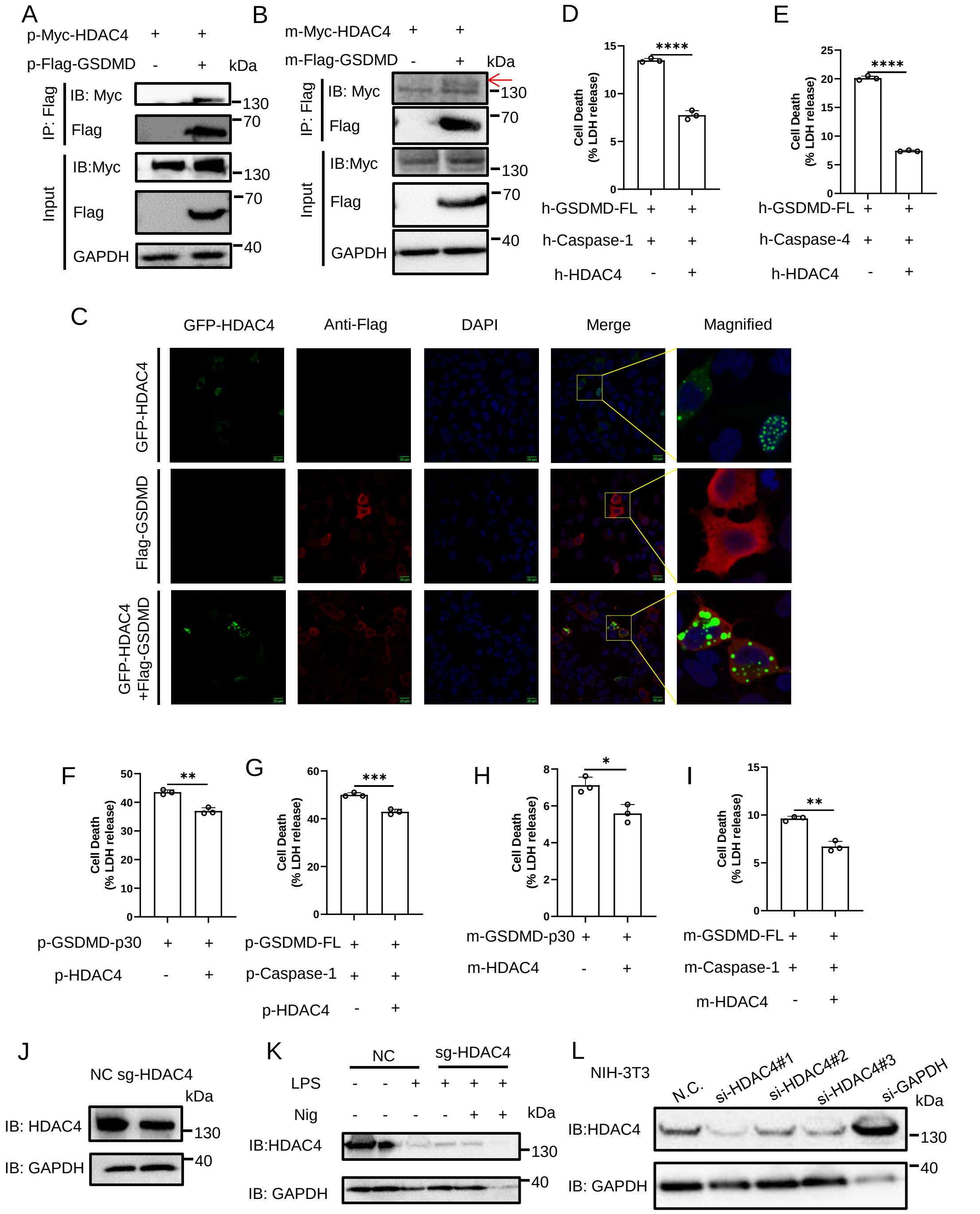

Supplementary Fig. 5 HDAC4 suppresses pyroptotic cell death. (A, B) IB of total cell lysates (input) and proteins immunoprecipitated with anti-Flag resin from HEK293T cells transfected with Myc-HDAC4 and Flag-GSDMD from pig (A) or mouse (B). **(C)** Immunofluorescence microscopy and nuclear staining (with the DNA-binding dye DAPI) of HEK293T cells transfected with expression plasmids for GFP-

HDAC4 and Flag-GSDMD. Scale bars, 20 μm. **(D, E)** LDH release of HEK293T cells transfected with GSDMD-FL and Caspase-1 or GSDMD-FL and Caspase-4, together with or without HDAC4. **(F-I)** LDH release of HEK293T cells transfected with GSDMD-p30 and GSDMD-FL and Caspase-1 from pig (F-G) or mouse (H-I), together with or without HDAC4. **(J)** IB of NC or sg-HDAC4 HKE293T cells. **(K)** IB of NC or sg-HDAC4 THP-1 cells treated with LPS and Nig. **(L)** NIH-3T3 cells were transfected with non-targeting control siRNA (si-NC) or siRNA targeting HDAC4 (si-HDAC4#1, #2, #3), or siRNA targeting GAPDH for 24 h. Cell lysates were analyzed by immunoblotting. ****stands for $P<0.0001$, ***stands for $P<0.001$, ** stands for $P<0.01$, * stands for $P<0.05$ and ns stands for no significant difference (unpaired t test). Data shown are mean ± SD from one representative experiments performed in triplicate.

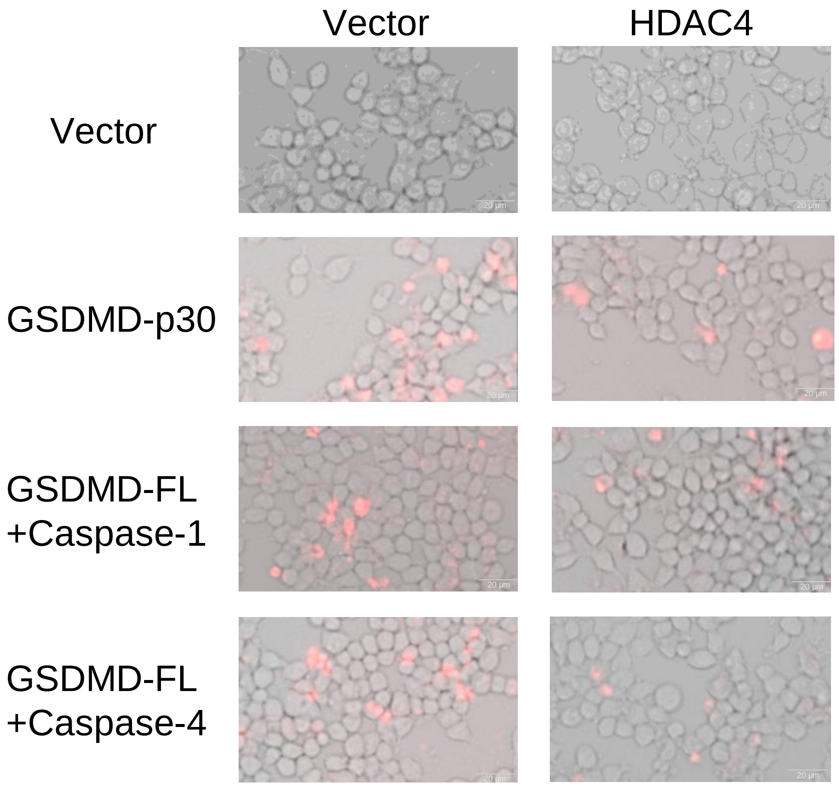

Supplementary Fig. 6 PI staining for Fig. 3F. PI staining of HEK293T cells after 24 h transfection with indicated plasmids. Scale bars, 200 µm.

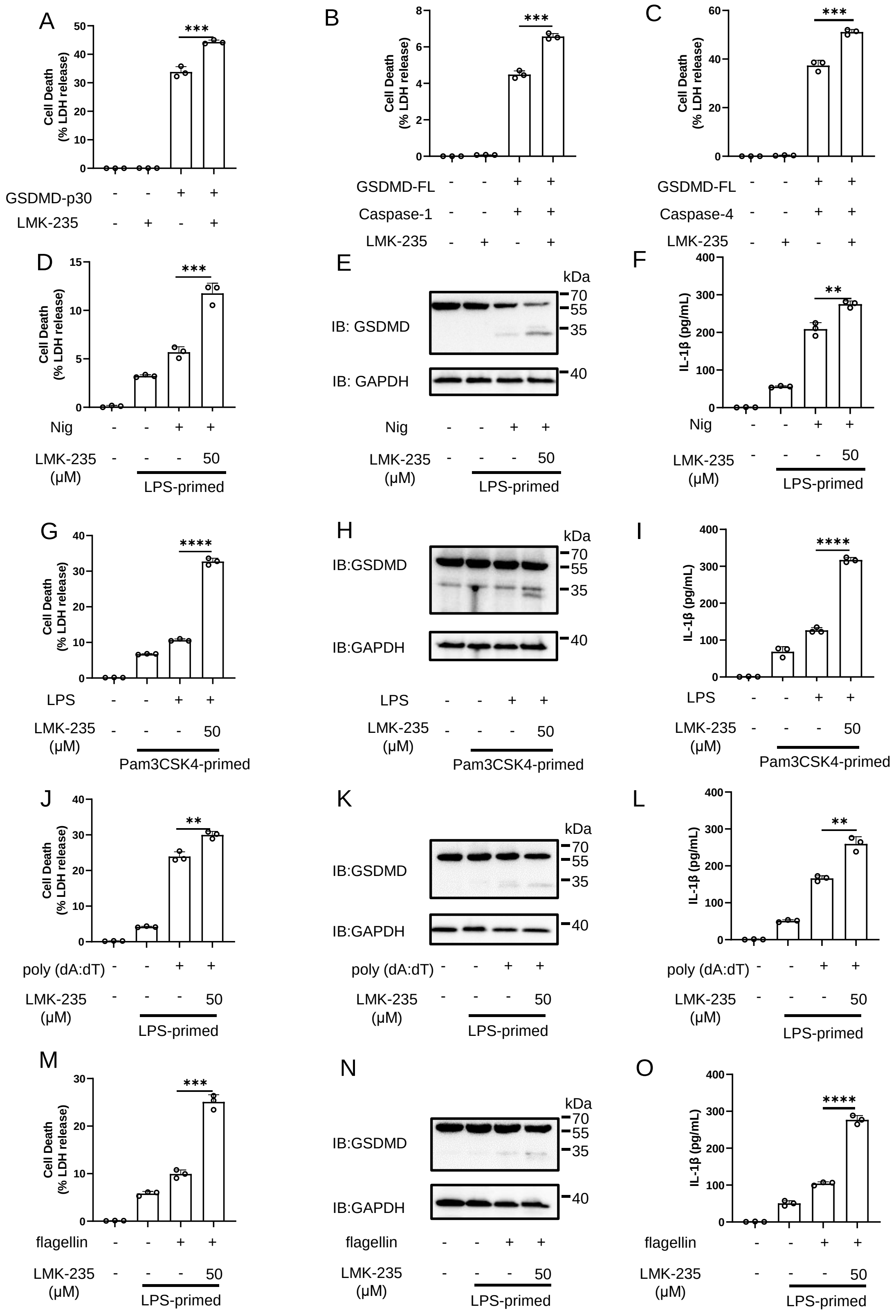

Supplementary Fig. 7 LMK-235-treatment promotes pyroptosis. (A-C) LDH release of HEK293T cells transfected with GSDMD-FL and Caspase-1 or GSDMD-

FL and Caspase-4, treated with or without LMK-235 (50 μM). **(D-F)** THP-1 cells were incubated with 500 ng/mL LPS and LMK-235 for 4 h and then another 1 h for 10 μM Nigericin. The supernatants were collected and analyzed by LDH release assay (D) and ELISA for IL-1β (F). Cell lysates were analyzed by immunoblotting (E). **(G-I)** PMA-differentiated THP-1 cells were primed with 250 ng/mL Pam3CSK4 and LMK-235 for 4 h, and then transfected with 2 μg/mL LPS for 6 h. The supernatants were collected and analyzed by LDH release assay (G) and ELISA for IL-1β (I). Cell lysates were analyzed by immunoblotting (H). **(J-L)** PMA-differentiated THP-1 cells were primed with 500 ng/mL LPS and LMK-235 for 4 h, and then transfected with 2 μg/mL poly(dA:dT) for 6 h. The supernatants were collected and analyzed by LDH release assay (J) and ELISA for IL-1β (L). Cell lysates were analyzed by immunoblotting (K). **(M-O)** PMA-differentiated THP-1 cells were primed with 500 ng/mL LPS and LMK-235 for 4 h, and then transfected with 2 μg/mL flagellin for 6 h. The supernatants were collected and analyzed by LDH release assay (M) and ELISA for IL-1β (O). Cell lysates were analyzed by immunoblotting (N). ****stands for $P<0.0001$, ***stands for $P<0.001$, ** stands for $P<0.01$ (unpaired t test). Data shown are mean ± SD from one representative experiments performed in triplicate.

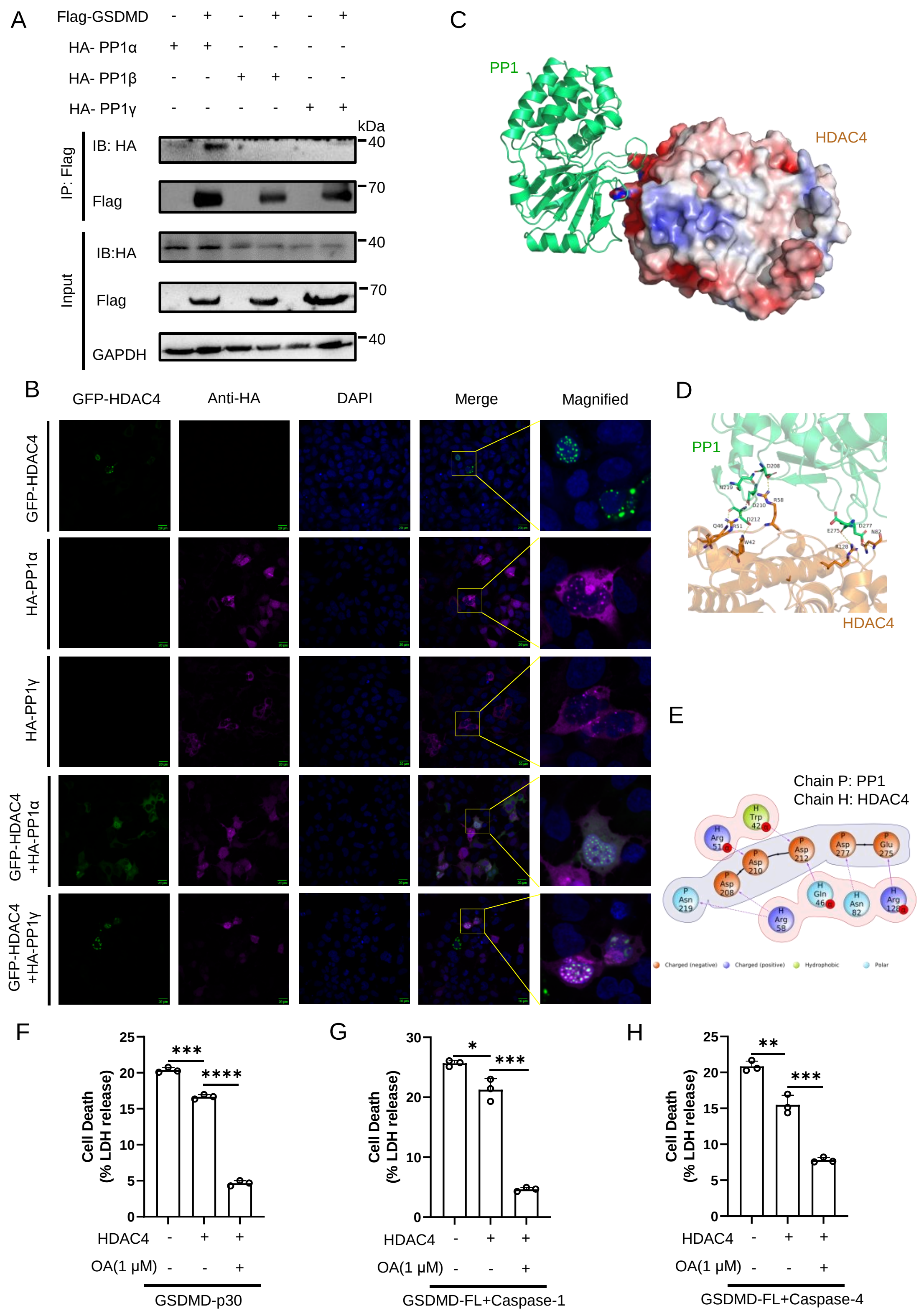

Supplementary Fig. 8 PP1 mediated dephosphorylation of HDAC4. (A) IB of total cell lysates (input) and proteins immunoprecipitated with anti-Flag resin from HEK293T cells transfected with Flag-HDAC4 and HA-PP1 subunits. **(B)**

Immunofluorescence microscopy and nuclear staining (with the DNA-binding dye DAPI) of HEK293T cells transfected with expression plasmids for GFP-HDAC4 and HA-PP1 subunits. Scale bars, 20 μm. **(C-E)** Structural model of the interaction of HDAC4 (PDB: 2VQW) and PP1 (PDB:4MOV). Hydrogen bonds are represented by yellow dotted lines in 3D display (D). Hydrogen bonds are represented by straight arrows or dashed lines in interaction 2D map (E). The docking models were analyzed using HDOCK Server. **(F-H)** LDH release of HEK293T cells co-transfected with GSDMD-p30 (F) or GSDMD-FL and Caspase-1 (G) or GSDMD-FL and Caspase-4 (H) and HDAC4, treated with or without OA (1μM). ****stands for $P<0.0001$, ***stands for $P<0.001$, ** stands for $P<0.01$ and * stands for $P<0.05$ (unpaired t test). Data shown are mean ± SD from one representative experiments performed in triplicate.

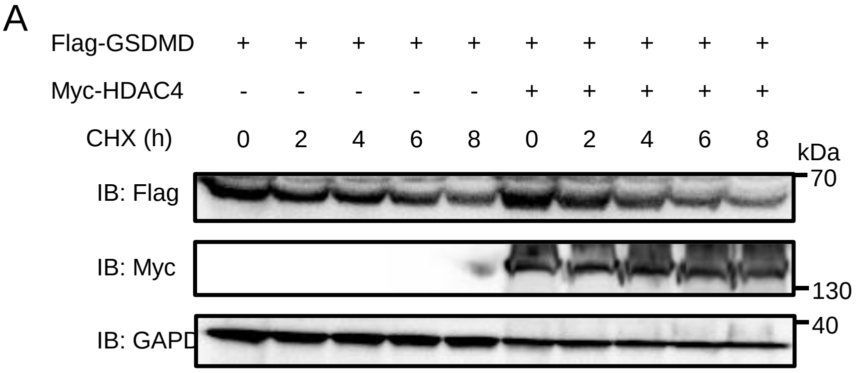

Supplementary Fig. 9 HDAC4 does not affect GSDMD degradation. (A) HEK293T cells were transfected with Flag-GSDMD together with or without Myc-HDAC4, then treated with cycloheximide (CHX) (25 μg/mL) for the indicated time points. Cell lysates were analyzed by immunoblotting. The result shown is representative of three independent experiments.

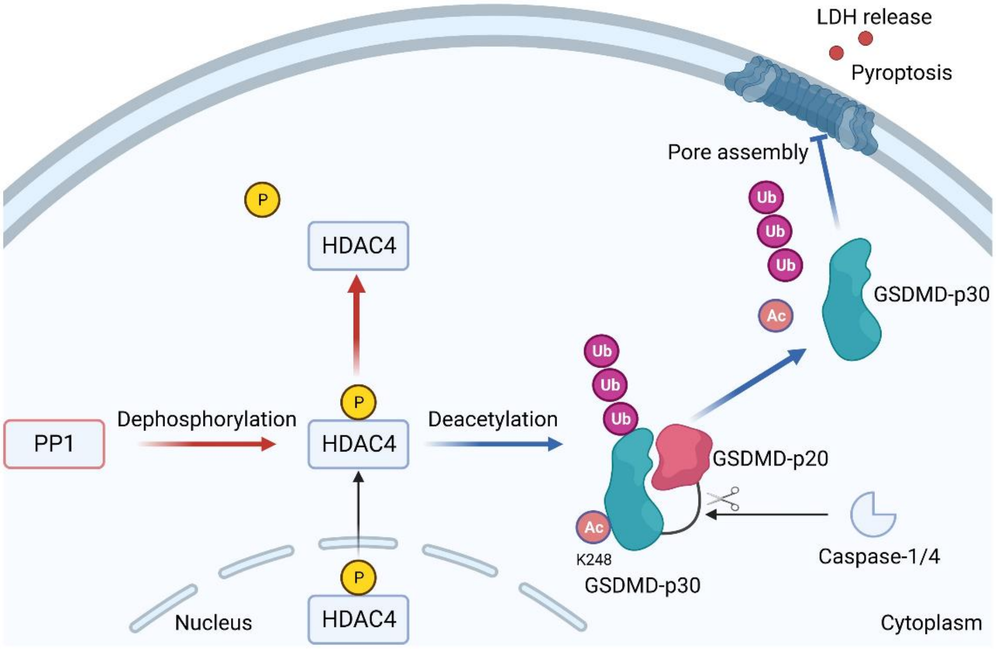

Supplementary Fig. 10 Diagram of HDAC4 regulates GSDMD-mediated pyroptosis.
