# Supplementary material for: Crosstalk of HDAC4, PP1, and GSDMD in controlling pyroptosis
Source: Cell Death Dis. 2024 Feb 7;15(2):115. doi: 10.1038/s41419-024-06505-z (PMC10850491; doi:10.1038/s41419-024-06505-z)
Supplement: Supplementary file 4 — Original western blots [file 41419_2024_6505_MOESM4_ESM.pptx]

## Slide 1
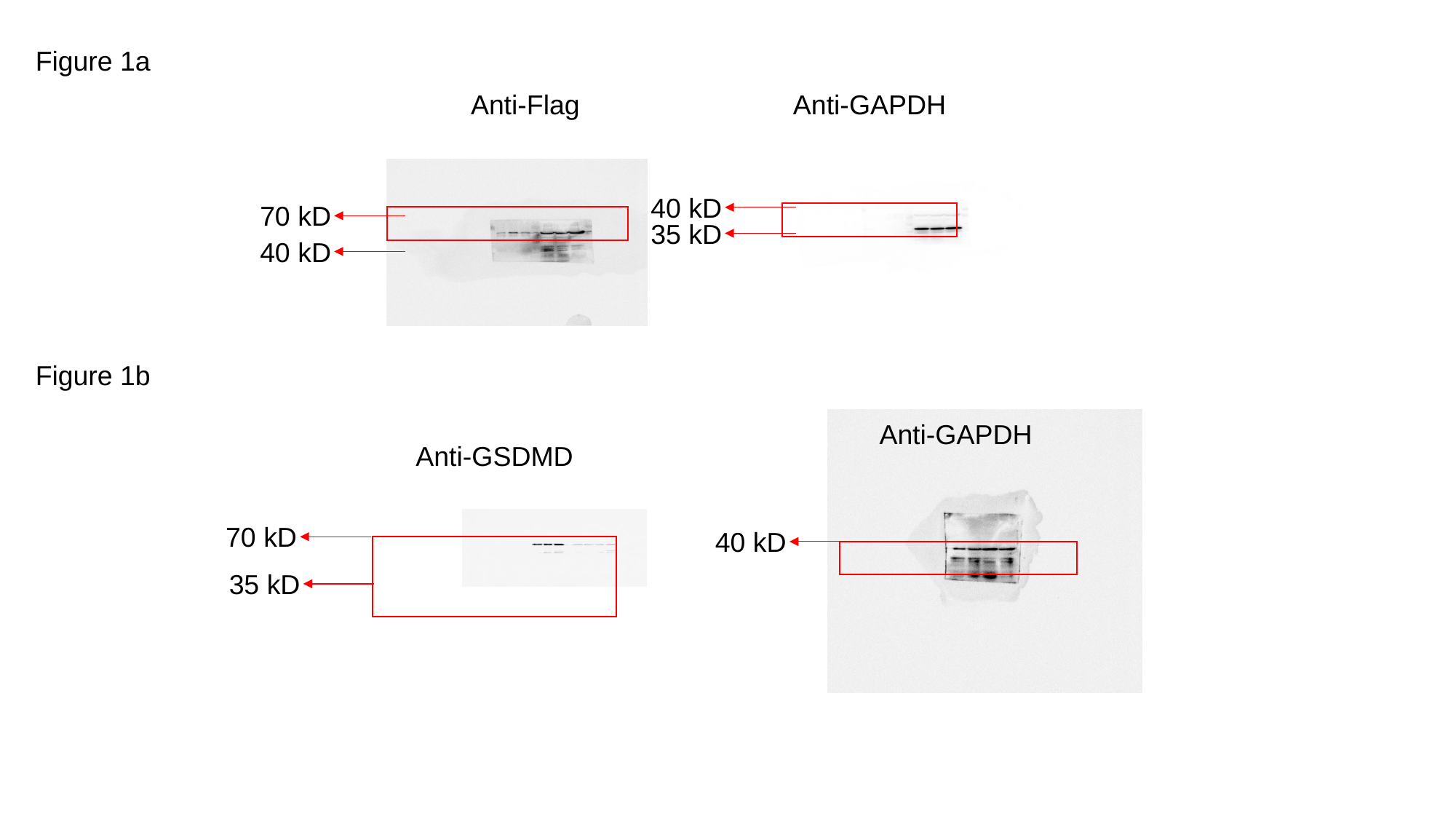

Figure 1a
Anti-Flag
Anti-GAPDH
40 kD
70 kD
35 kD
40 kD
Figure 1b
Anti-GAPDH
Anti-GSDMD
70 kD
40 kD
35 kD

## Slide 2
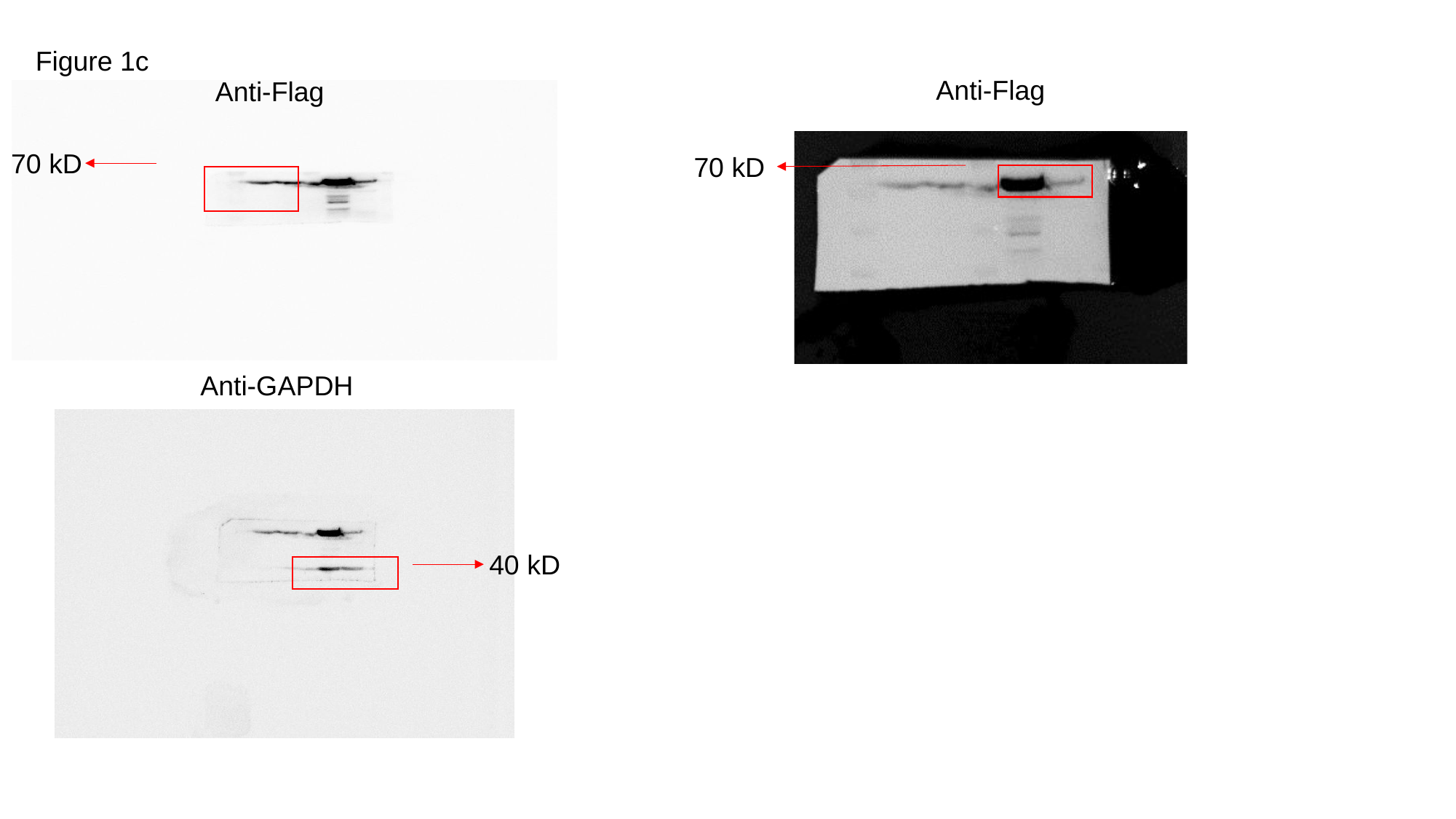

Figure 1c
Anti-Flag
Anti-Flag
70 kD
70 kD
Anti-GAPDH
40 kD

## Slide 3
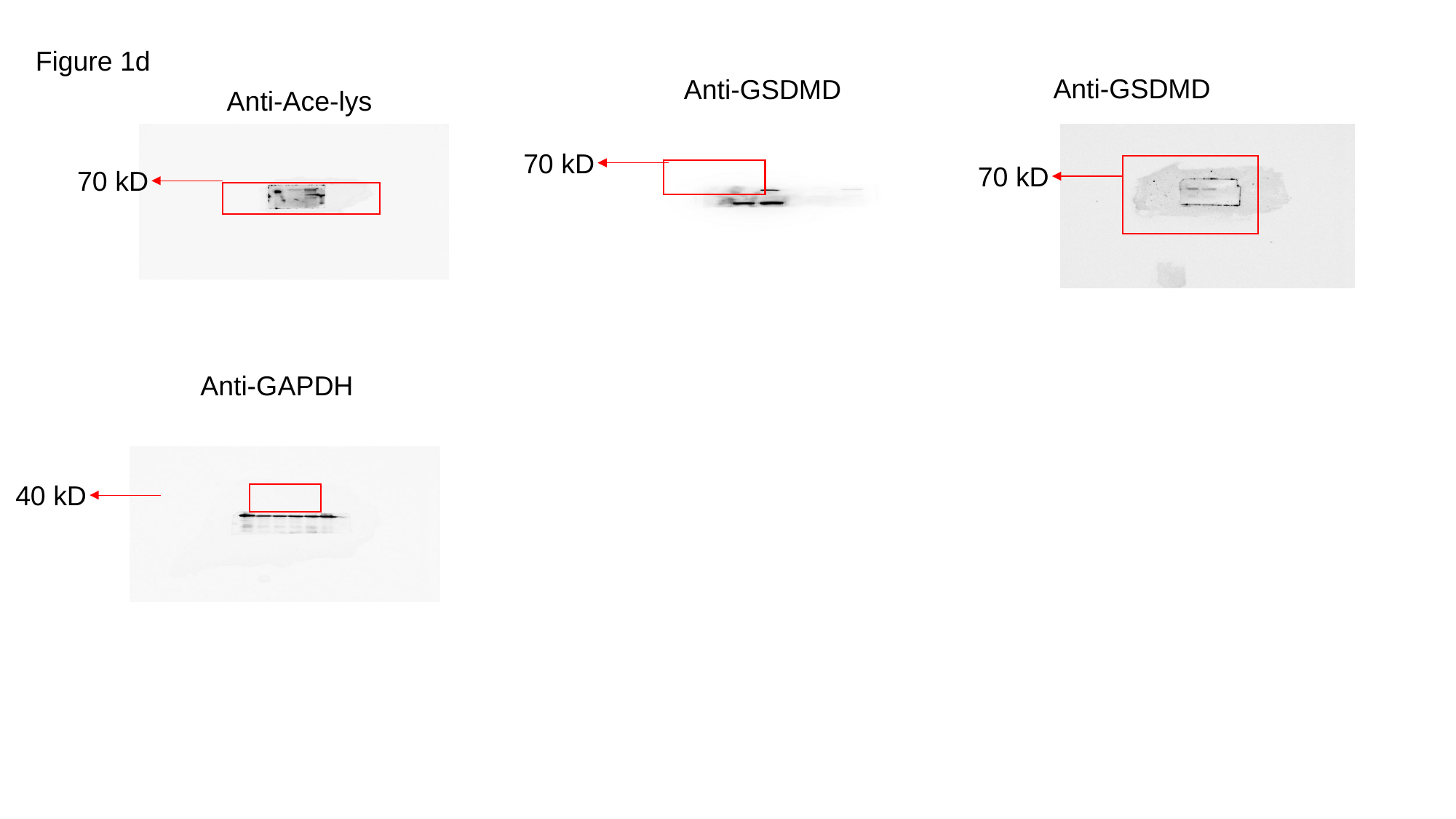

Figure 1d
Anti-GSDMD
Anti-GSDMD
Anti-Ace-lys
70 kD
70 kD
70 kD
Anti-GAPDH
40 kD

## Slide 4
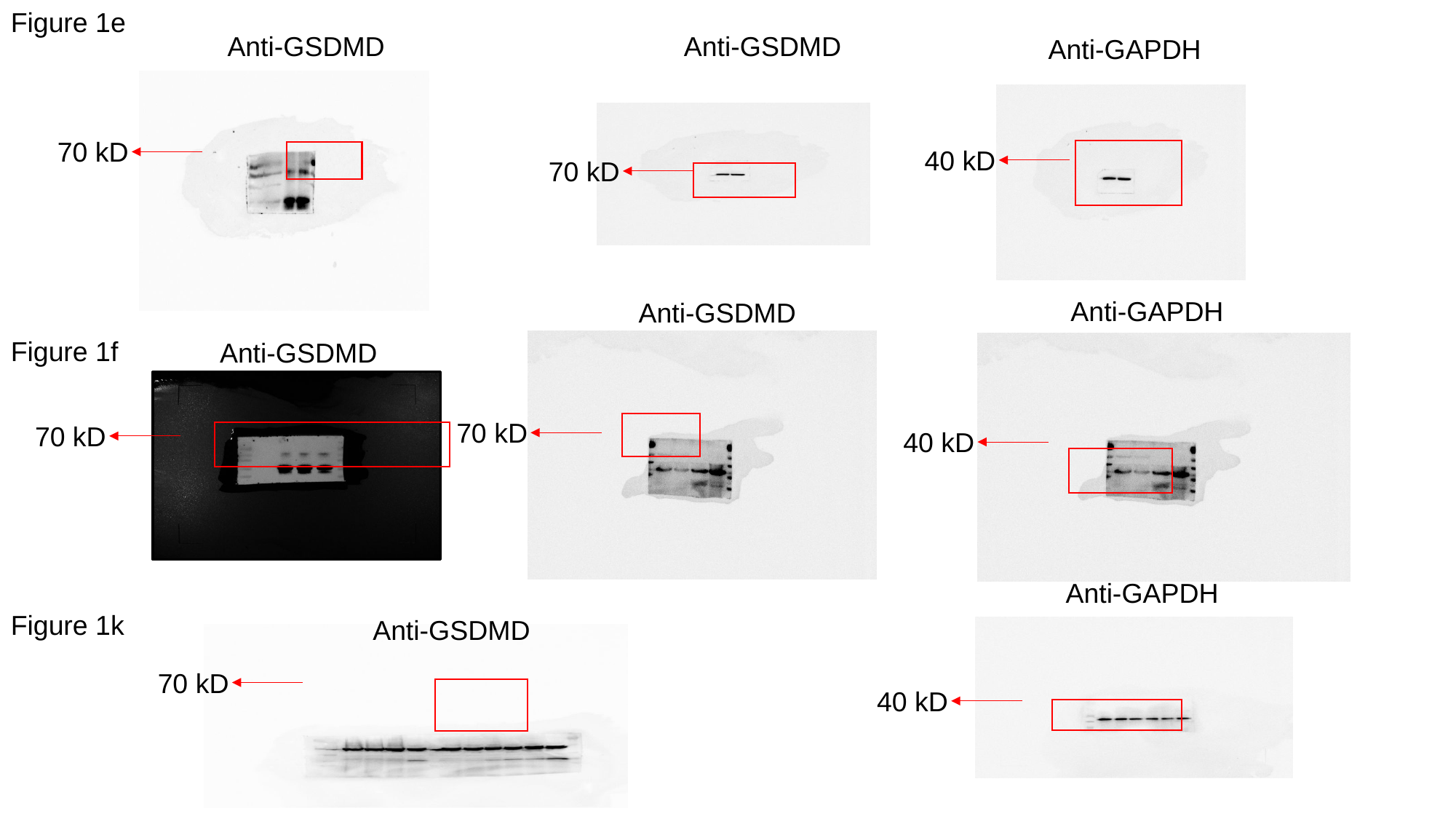

Figure 1e
Anti-GSDMD
Anti-GSDMD
Anti-GAPDH
70 kD
40 kD
70 kD
Anti-GAPDH
Anti-GSDMD
Figure 1f
Anti-GSDMD
70 kD
70 kD
40 kD
Anti-GAPDH
Figure 1k
Anti-GSDMD
70 kD
40 kD

## Slide 5
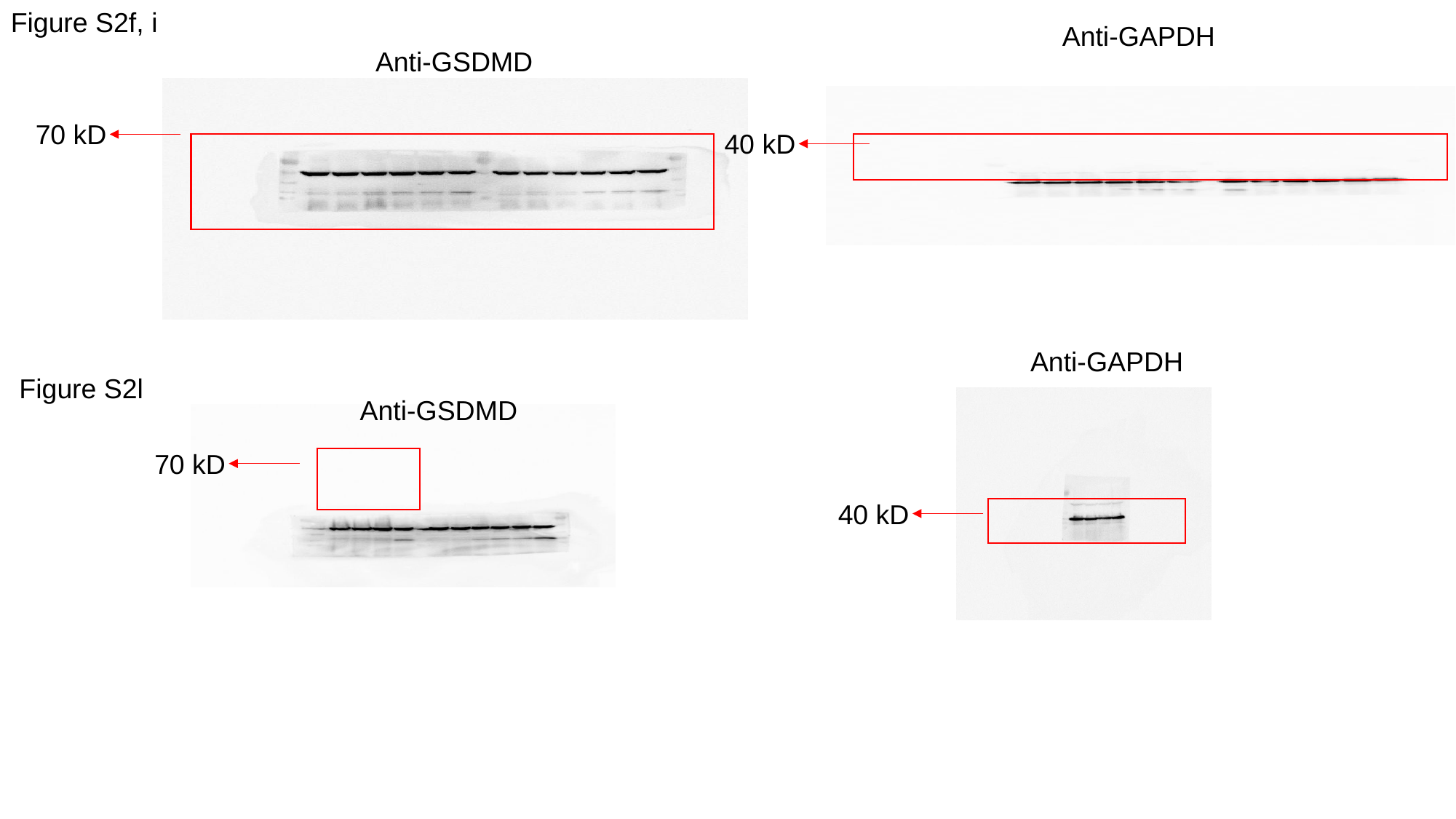

Figure S2f, i
Anti-GAPDH
Anti-GSDMD
70 kD
40 kD
Anti-GAPDH
Figure S2l
Anti-GSDMD
70 kD
40 kD

## Slide 6
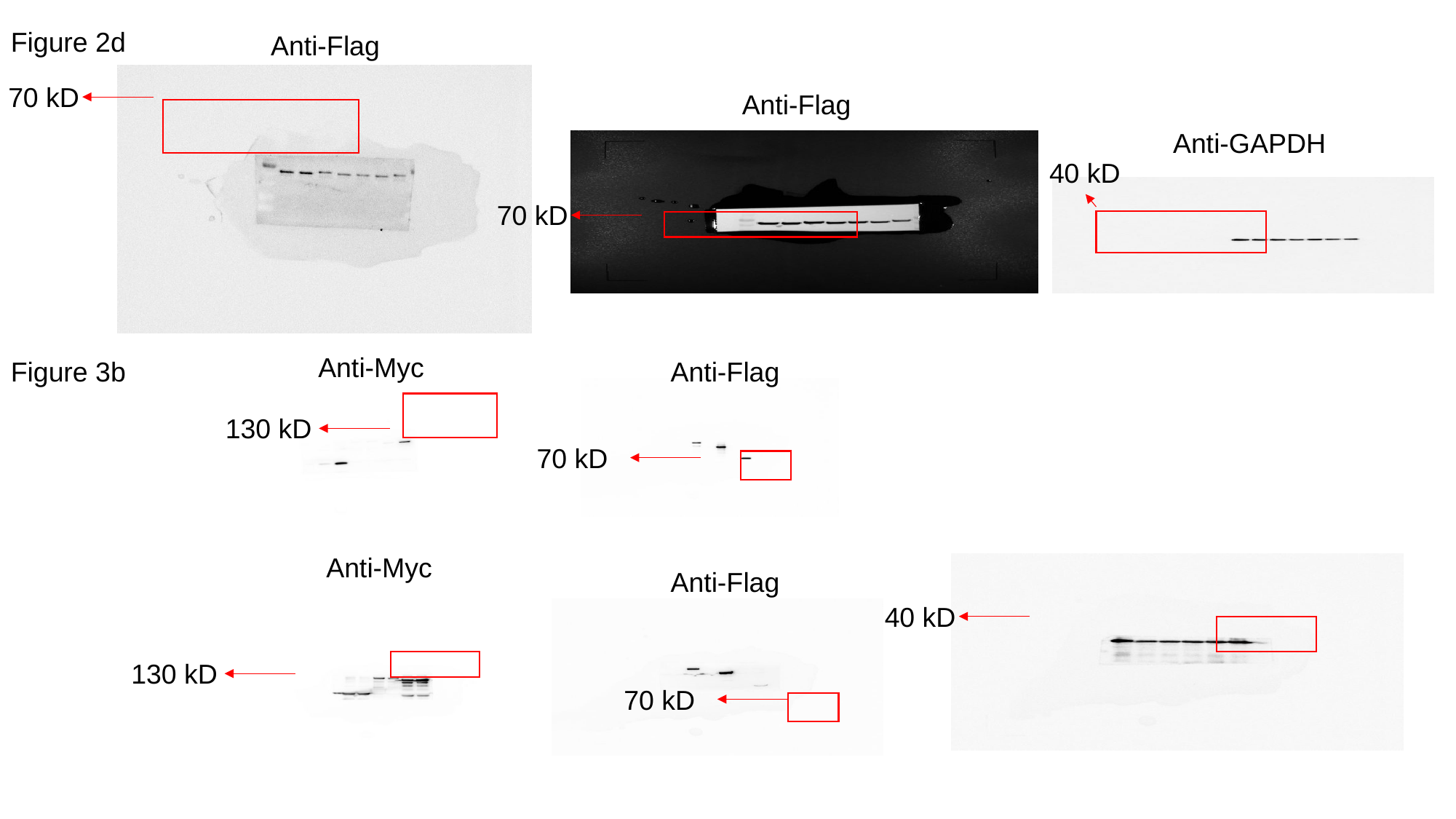

Figure 2d
Anti-Flag
70 kD
Anti-Flag
Anti-GAPDH
40 kD
70 kD
Anti-Myc
Figure 3b
Anti-Flag
130 kD
70 kD
Anti-Myc
Anti-Flag
40 kD
130 kD
70 kD

## Slide 7
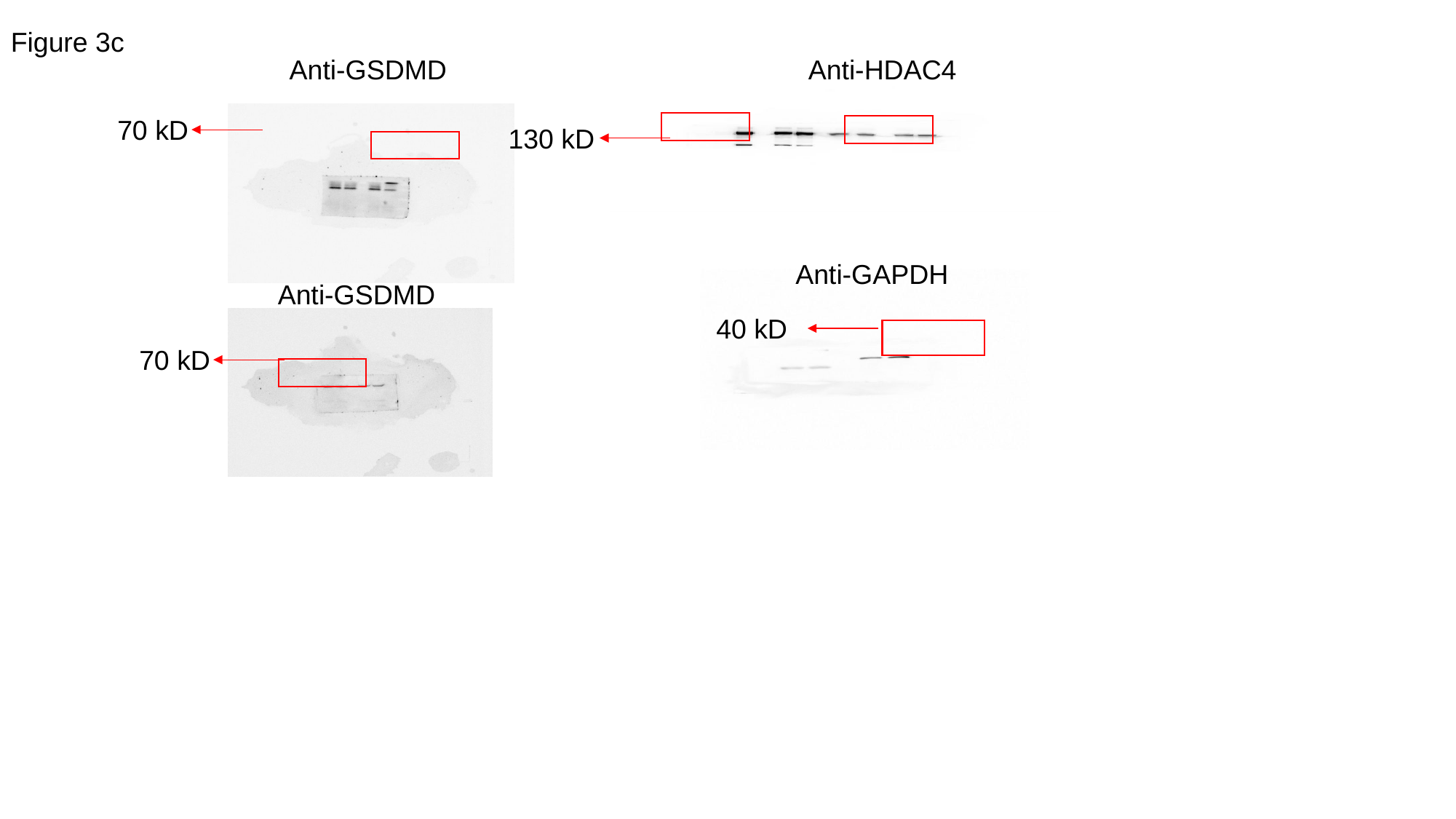

Figure 3c
Anti-GSDMD
Anti-HDAC4
70 kD
130 kD
Anti-GAPDH
Anti-GSDMD
40 kD
70 kD

## Slide 8
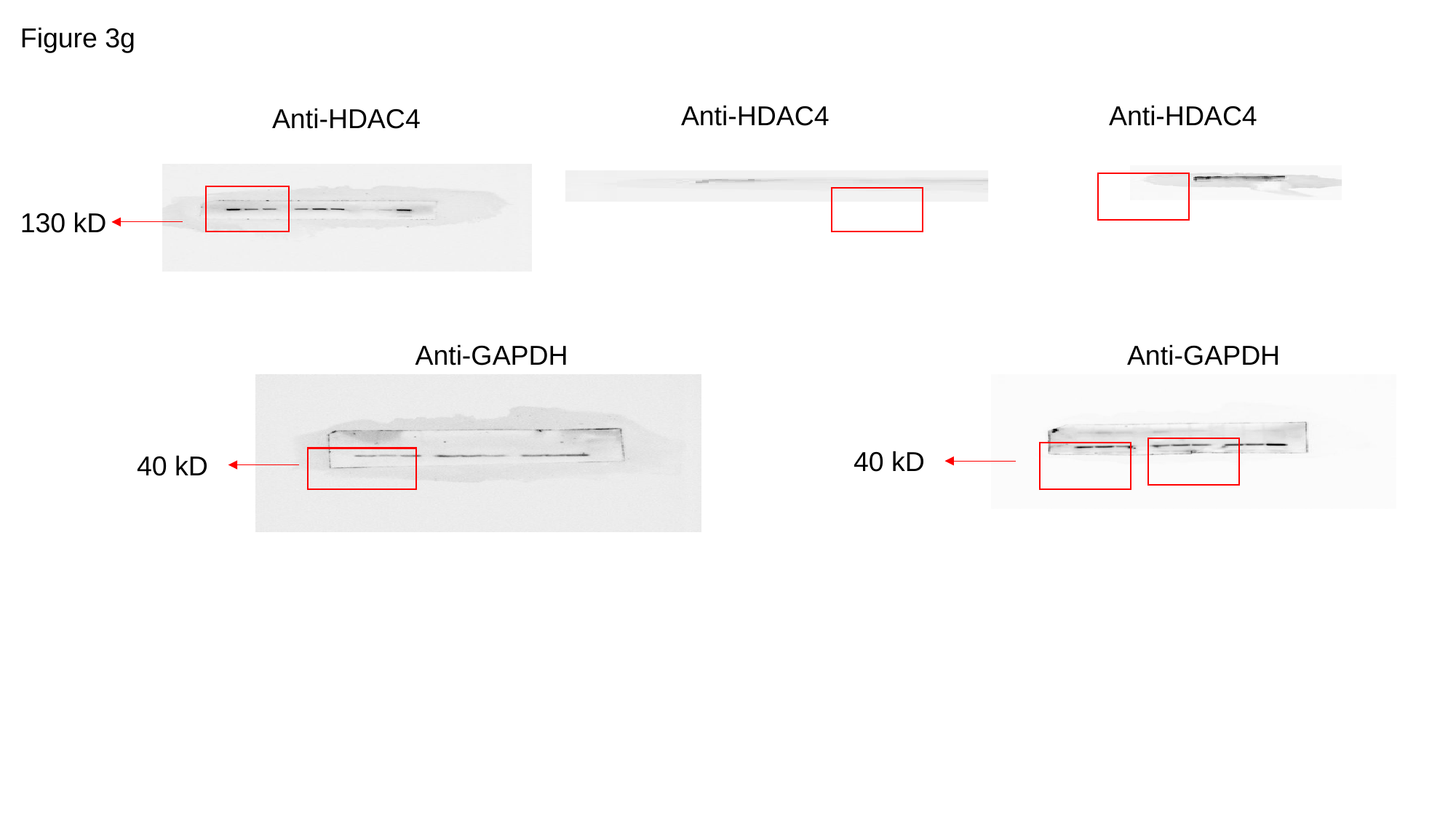

Figure 3g
Anti-HDAC4
Anti-HDAC4
Anti-HDAC4
130 kD
Anti-GAPDH
Anti-GAPDH
40 kD
40 kD

## Slide 9
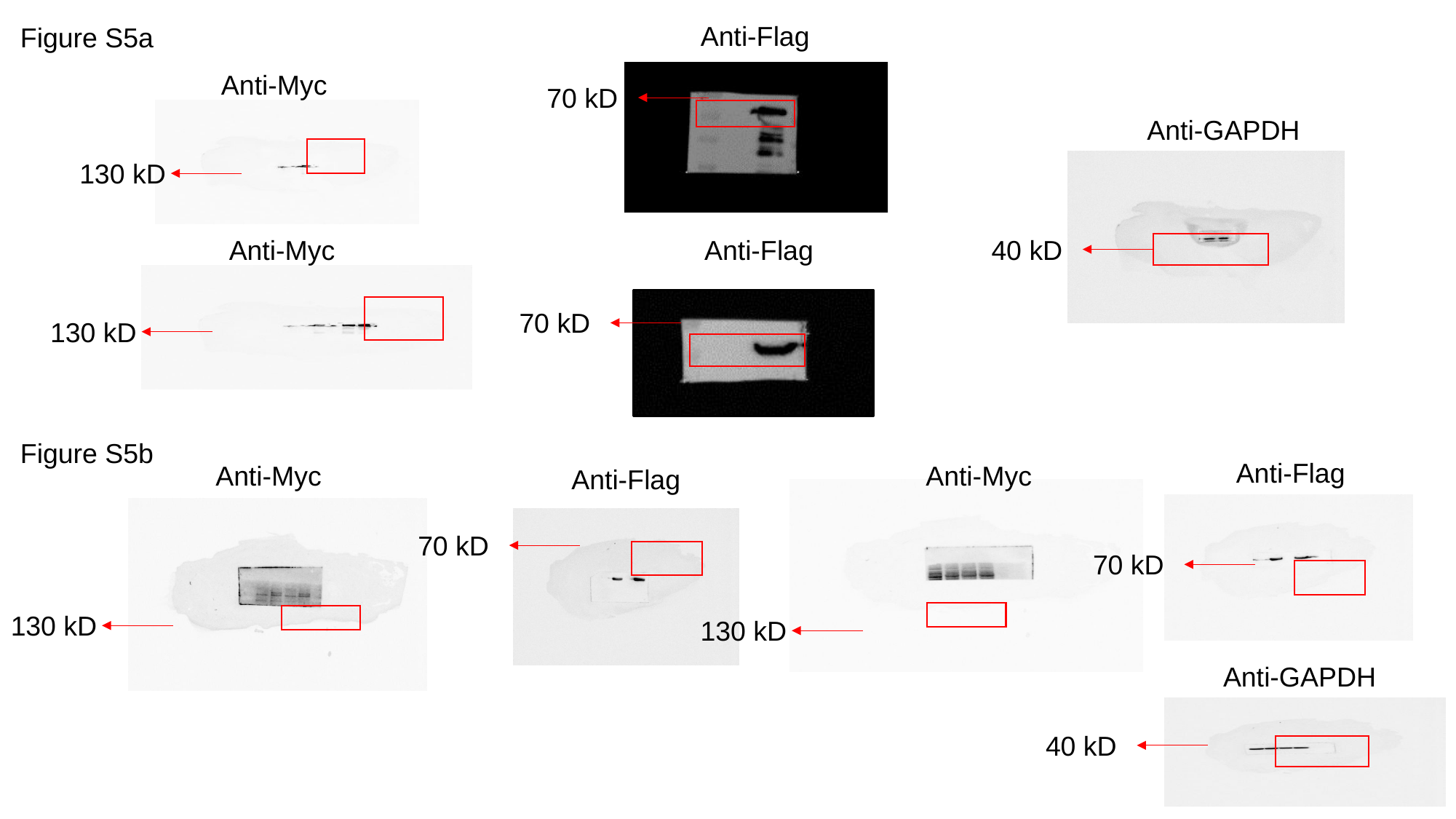

Anti-Flag
Figure S5a
Anti-Myc
70 kD
Anti-GAPDH
130 kD
Anti-Myc
Anti-Flag
40 kD
70 kD
130 kD
Figure S5b
Anti-Flag
Anti-Myc
Anti-Myc
Anti-Flag
70 kD
70 kD
130 kD
130 kD
Anti-GAPDH
40 kD

## Slide 10
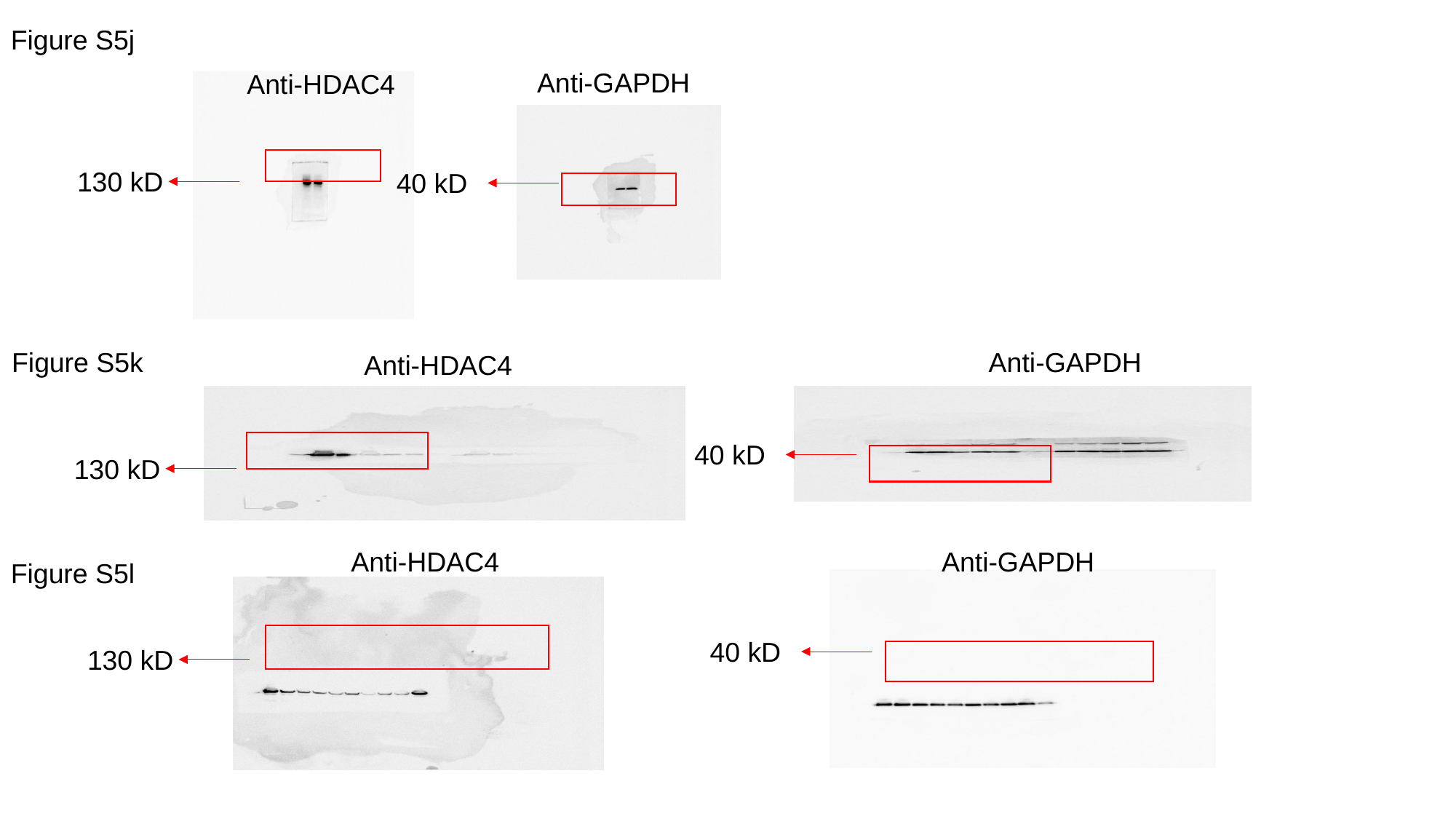

Figure S5j
Anti-GAPDH
Anti-HDAC4
130 kD
40 kD
Figure S5k
Anti-GAPDH
Anti-HDAC4
40 kD
130 kD
Anti-HDAC4
Anti-GAPDH
Figure S5l
40 kD
130 kD

## Slide 11
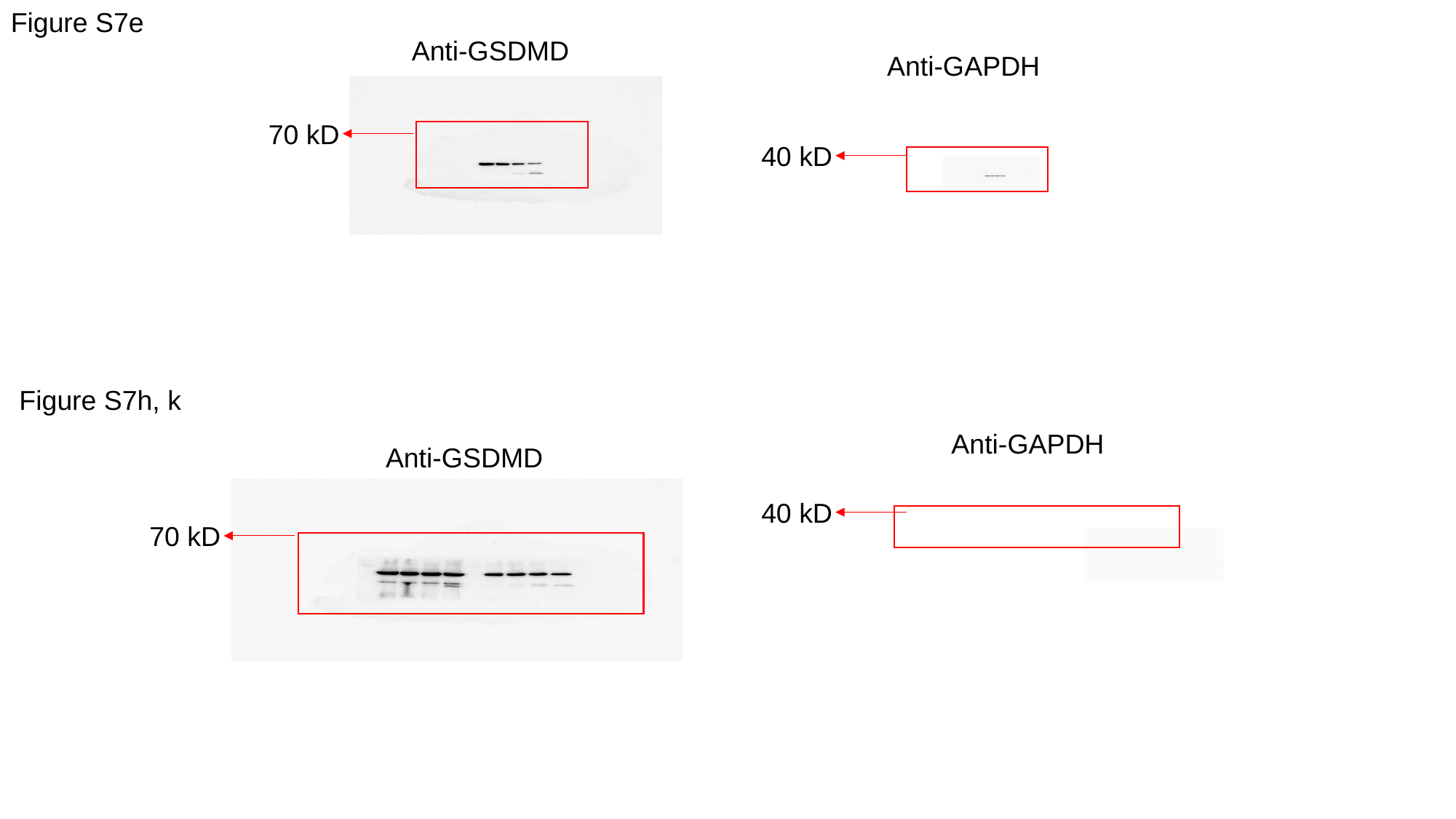

Figure S7e
Anti-GSDMD
Anti-GAPDH
70 kD
40 kD
Figure S7h, k
Anti-GAPDH
Anti-GSDMD
40 kD
70 kD

## Slide 12
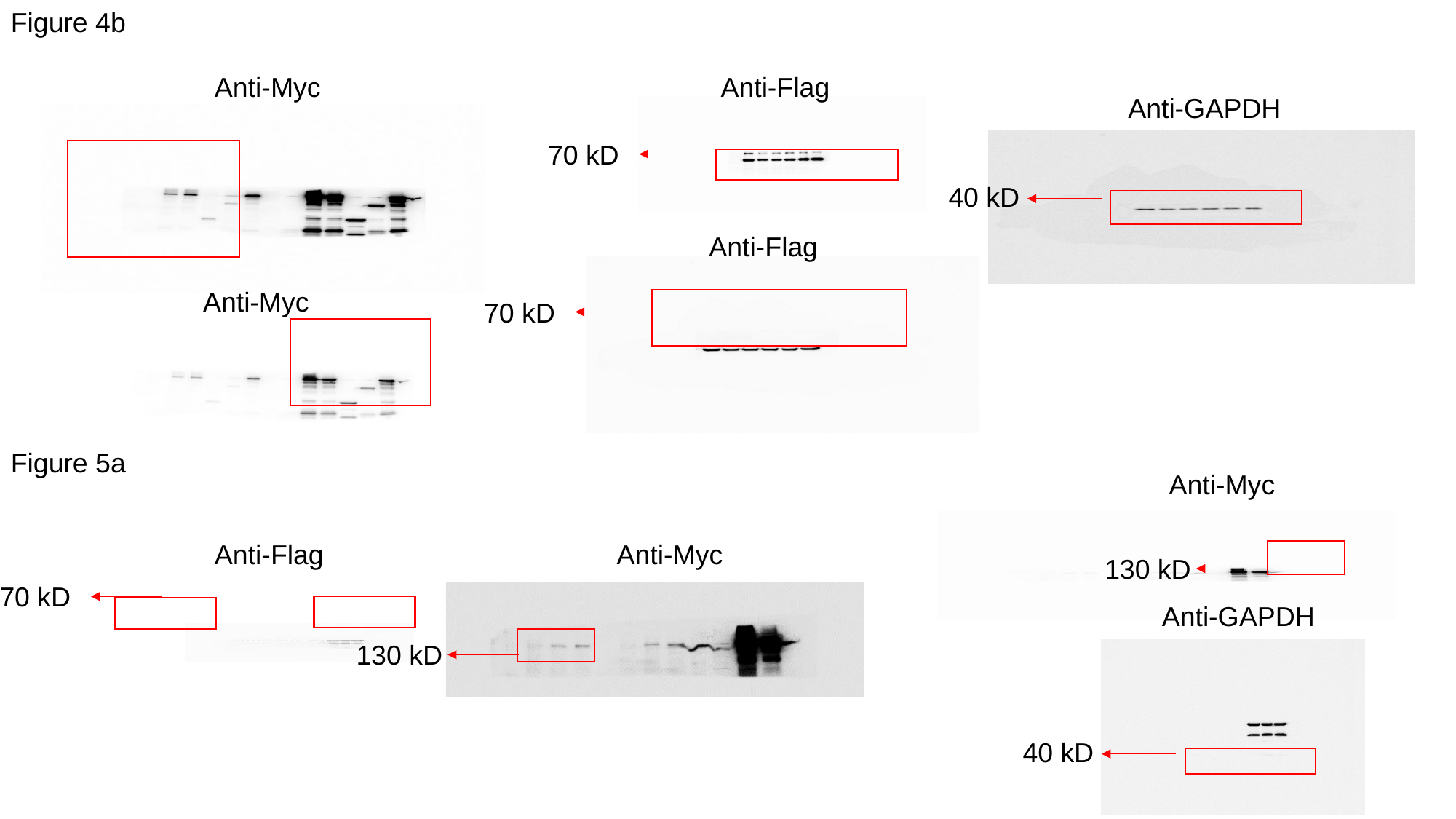

Figure 4b
Anti-Myc
Anti-Flag
Anti-GAPDH
70 kD
40 kD
Anti-Flag
Anti-Myc
70 kD
Figure 5a
Anti-Myc
Anti-Flag
Anti-Myc
130 kD
70 kD
Anti-GAPDH
130 kD
40 kD

## Slide 13
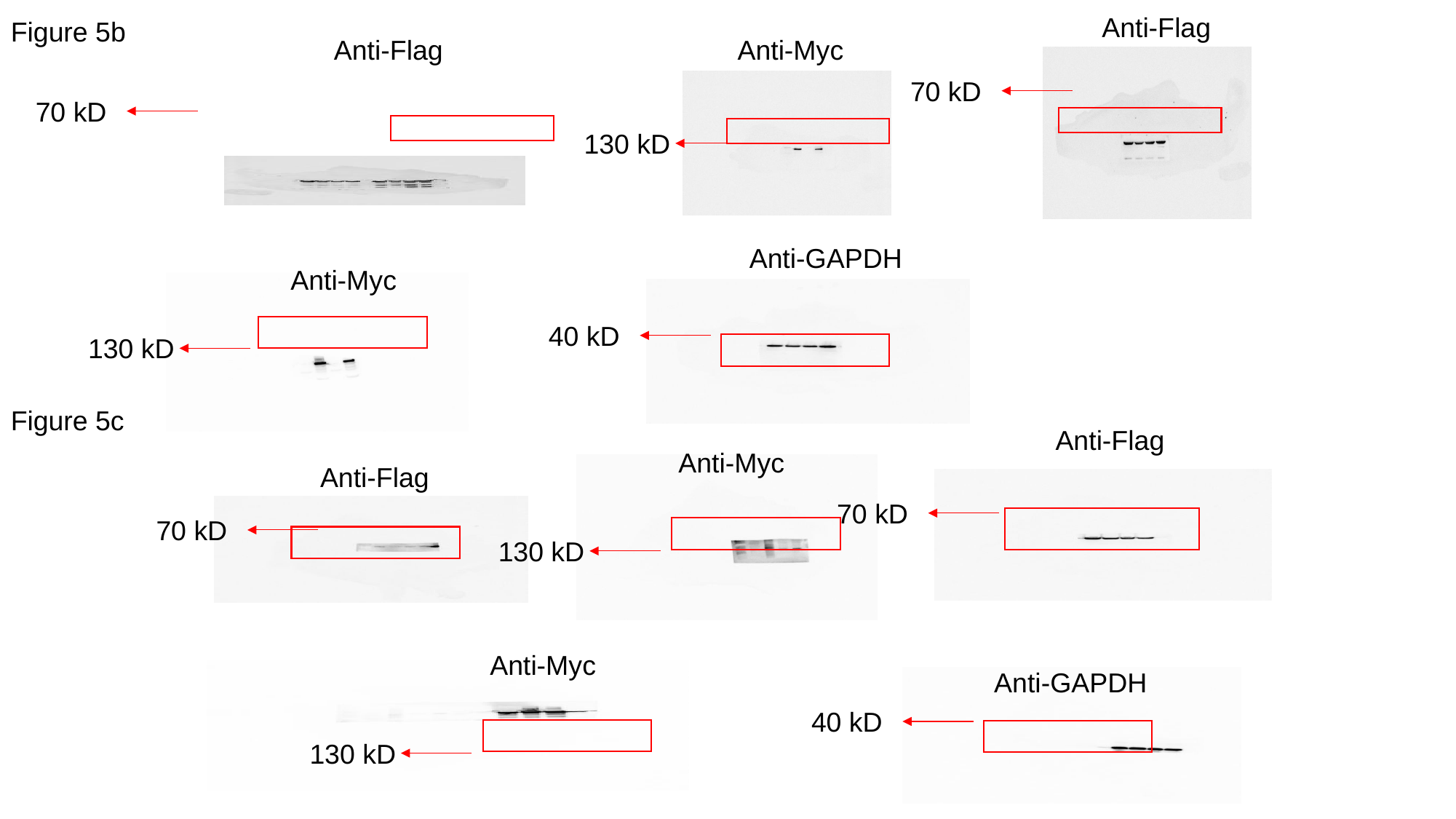

Anti-Flag
Figure 5b
Anti-Flag
Anti-Myc
70 kD
70 kD
130 kD
Anti-GAPDH
Anti-Myc
40 kD
130 kD
Figure 5c
Anti-Flag
Anti-Myc
Anti-Flag
70 kD
70 kD
130 kD
Anti-Myc
Anti-GAPDH
40 kD
130 kD

## Slide 14
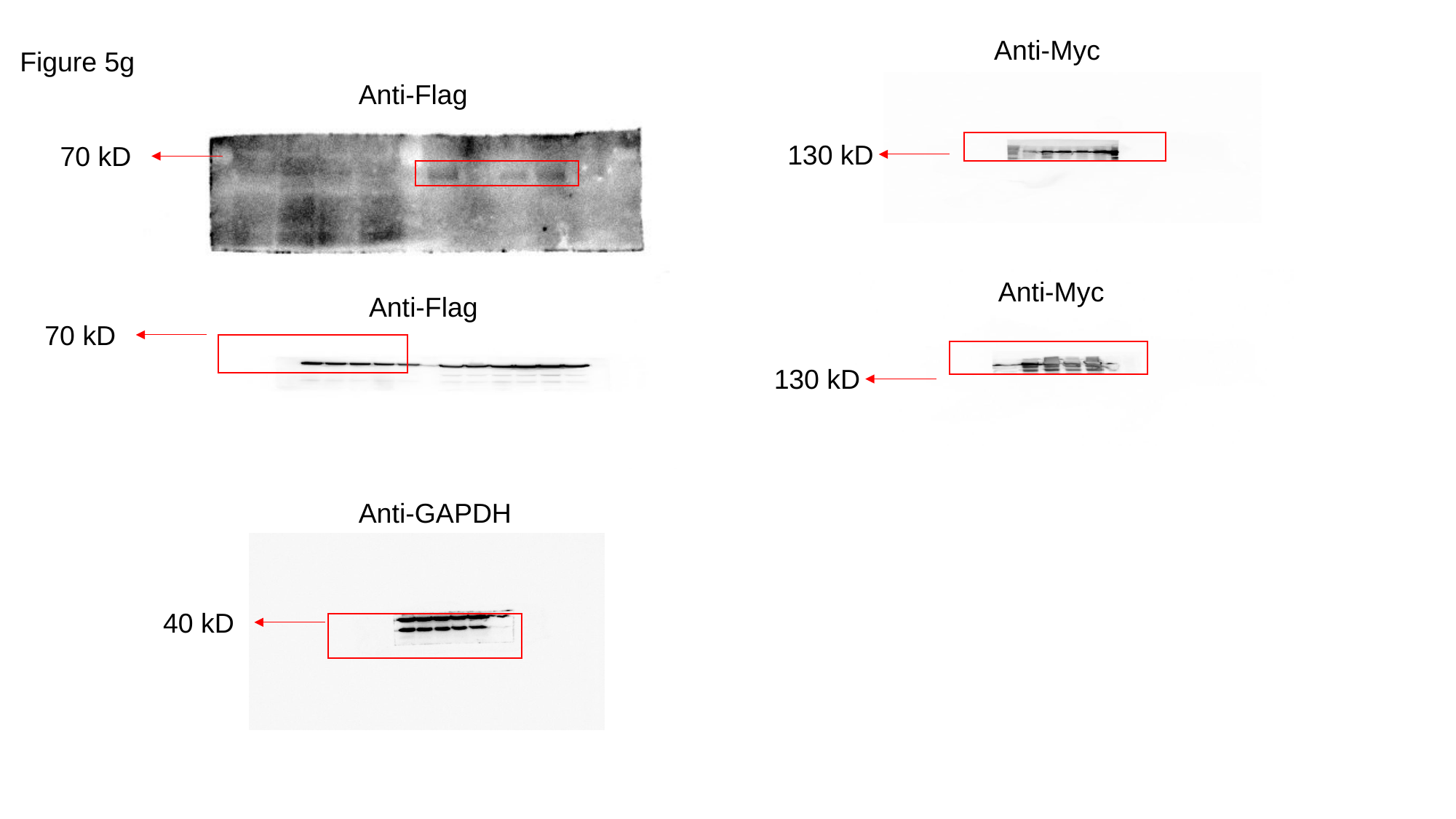

Anti-Myc
Figure 5g
Anti-Flag
130 kD
70 kD
Anti-Myc
Anti-Flag
70 kD
130 kD
Anti-GAPDH
40 kD

## Slide 15
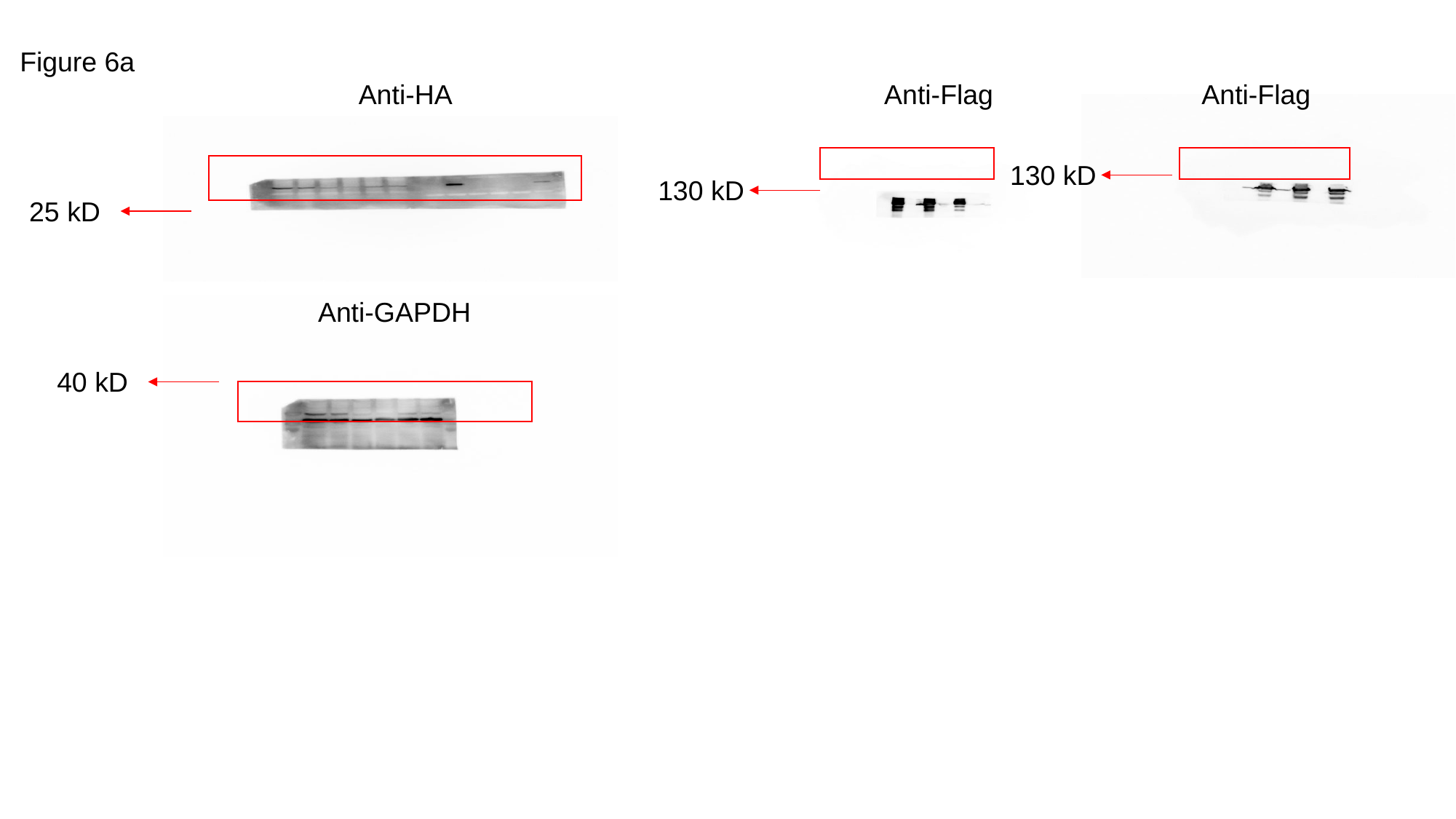

Figure 6a
Anti-HA
Anti-Flag
Anti-Flag
130 kD
130 kD
25 kD
Anti-GAPDH
40 kD

## Slide 16
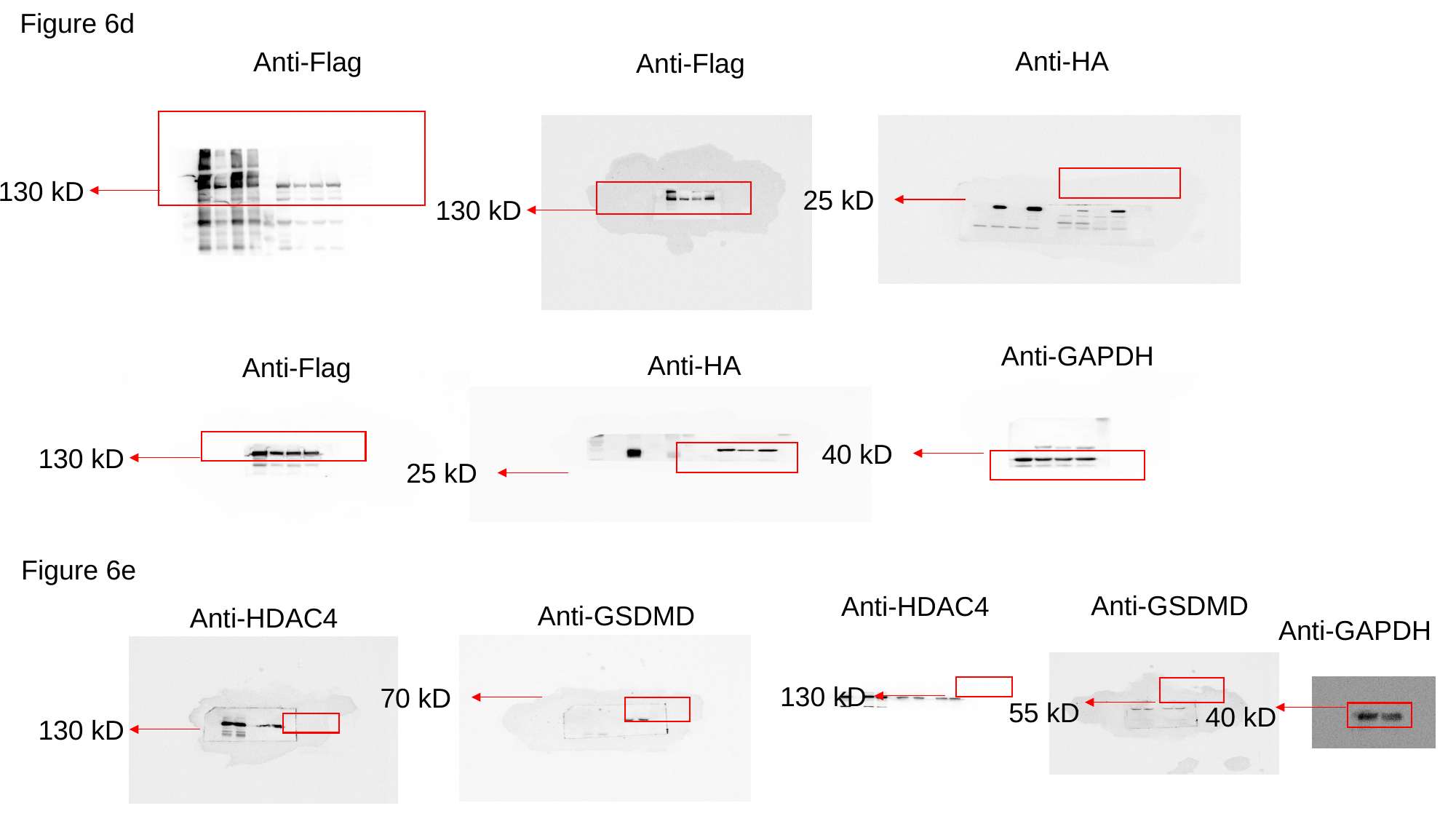

Figure 6d
Anti-HA
Anti-Flag
Anti-Flag
130 kD
25 kD
130 kD
Anti-GAPDH
Anti-HA
Anti-Flag
40 kD
130 kD
25 kD
Figure 6e
Anti-GSDMD
Anti-HDAC4
Anti-GSDMD
Anti-HDAC4
Anti-GAPDH
130 kD
70 kD
55 kD
40 kD
130 kD

## Slide 17
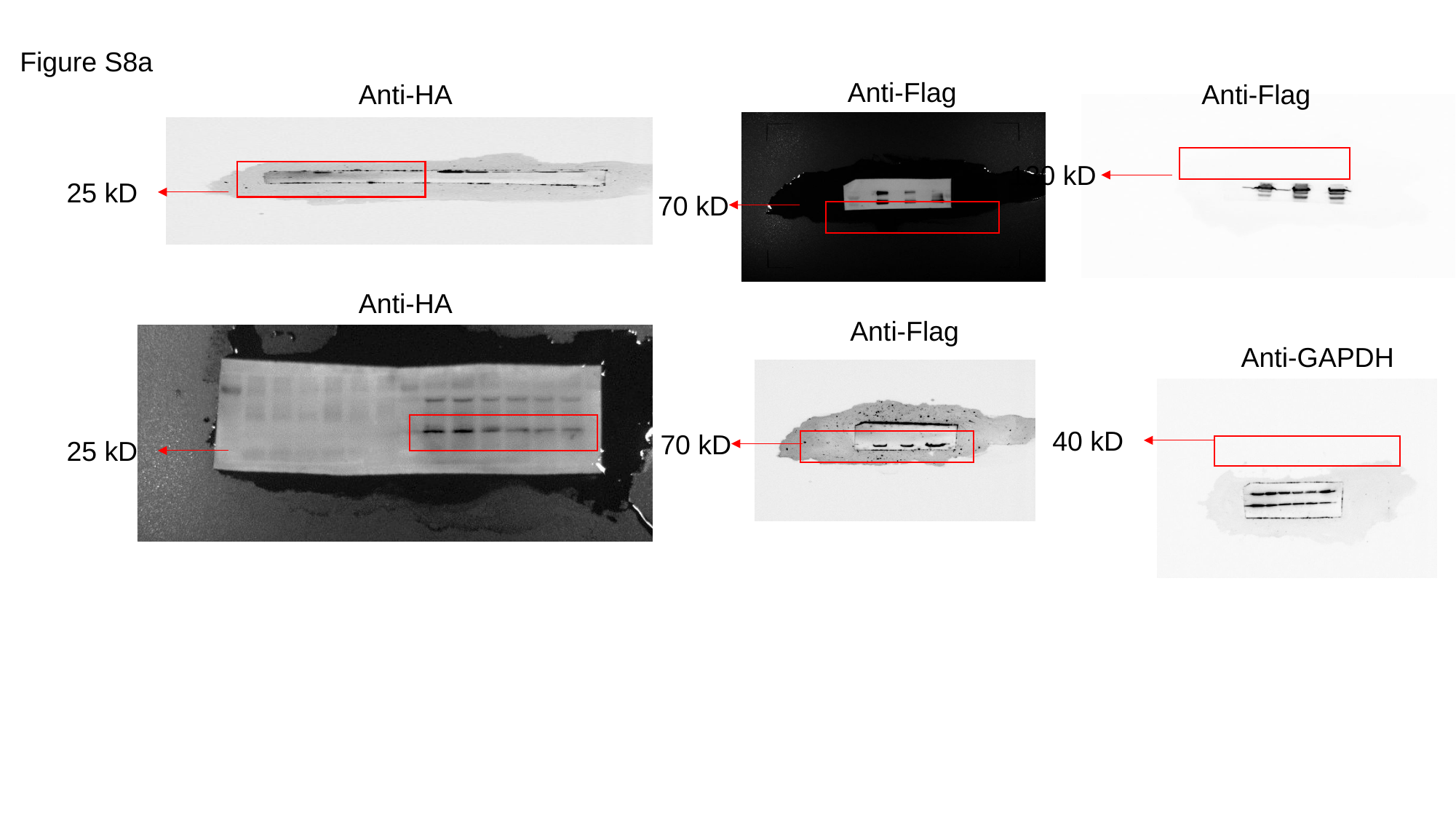

Figure S8a
Anti-Flag
Anti-HA
Anti-Flag
130 kD
25 kD
70 kD
Anti-HA
Anti-Flag
Anti-GAPDH
40 kD
70 kD
25 kD

## Slide 18
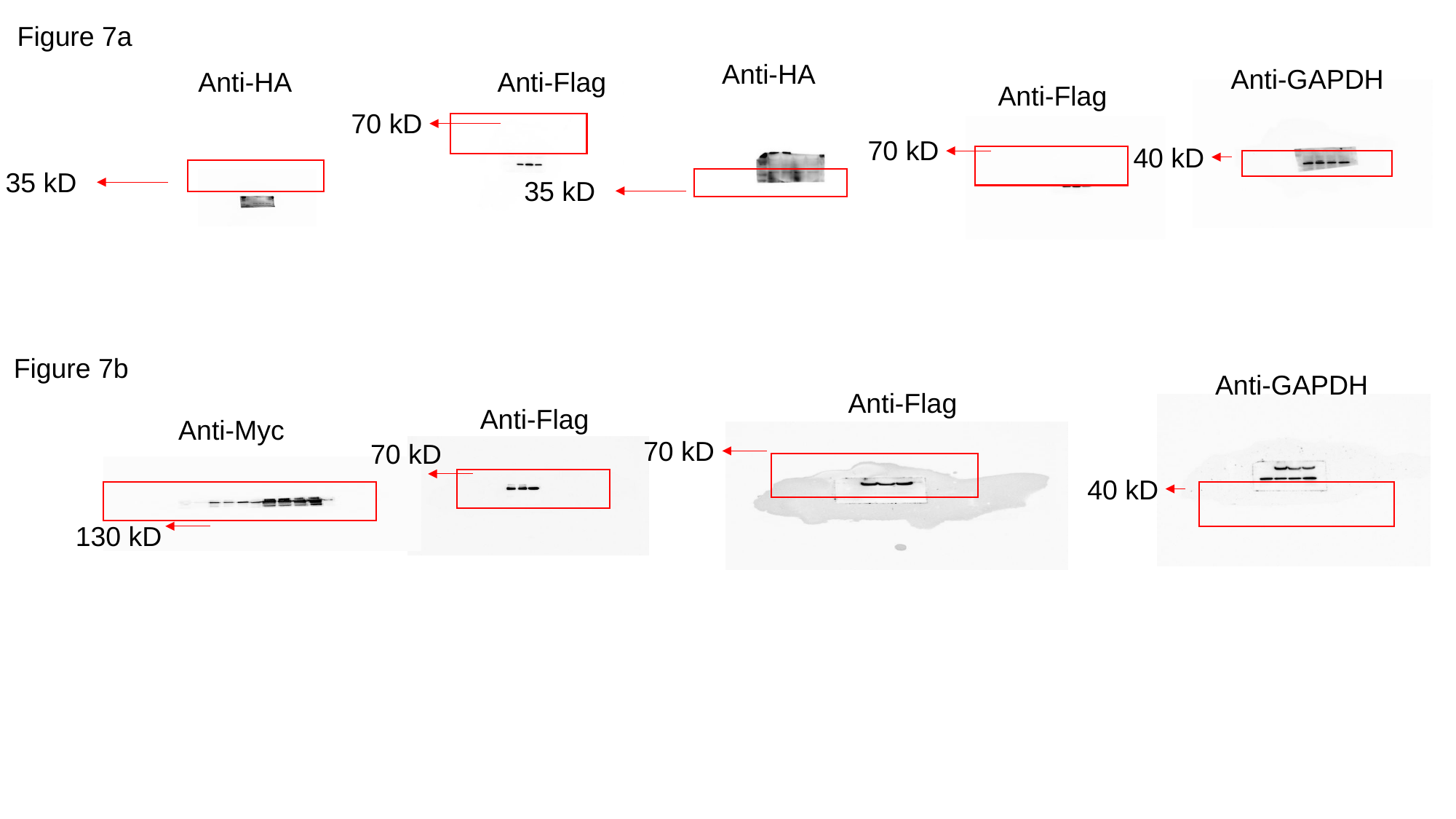

Figure 7a
Anti-HA
Anti-GAPDH
Anti-HA
Anti-Flag
Anti-Flag
70 kD
70 kD
40 kD
35 kD
35 kD
Figure 7b
Anti-GAPDH
Anti-Flag
Anti-Flag
Anti-Myc
70 kD
70 kD
40 kD
130 kD

## Slide 19
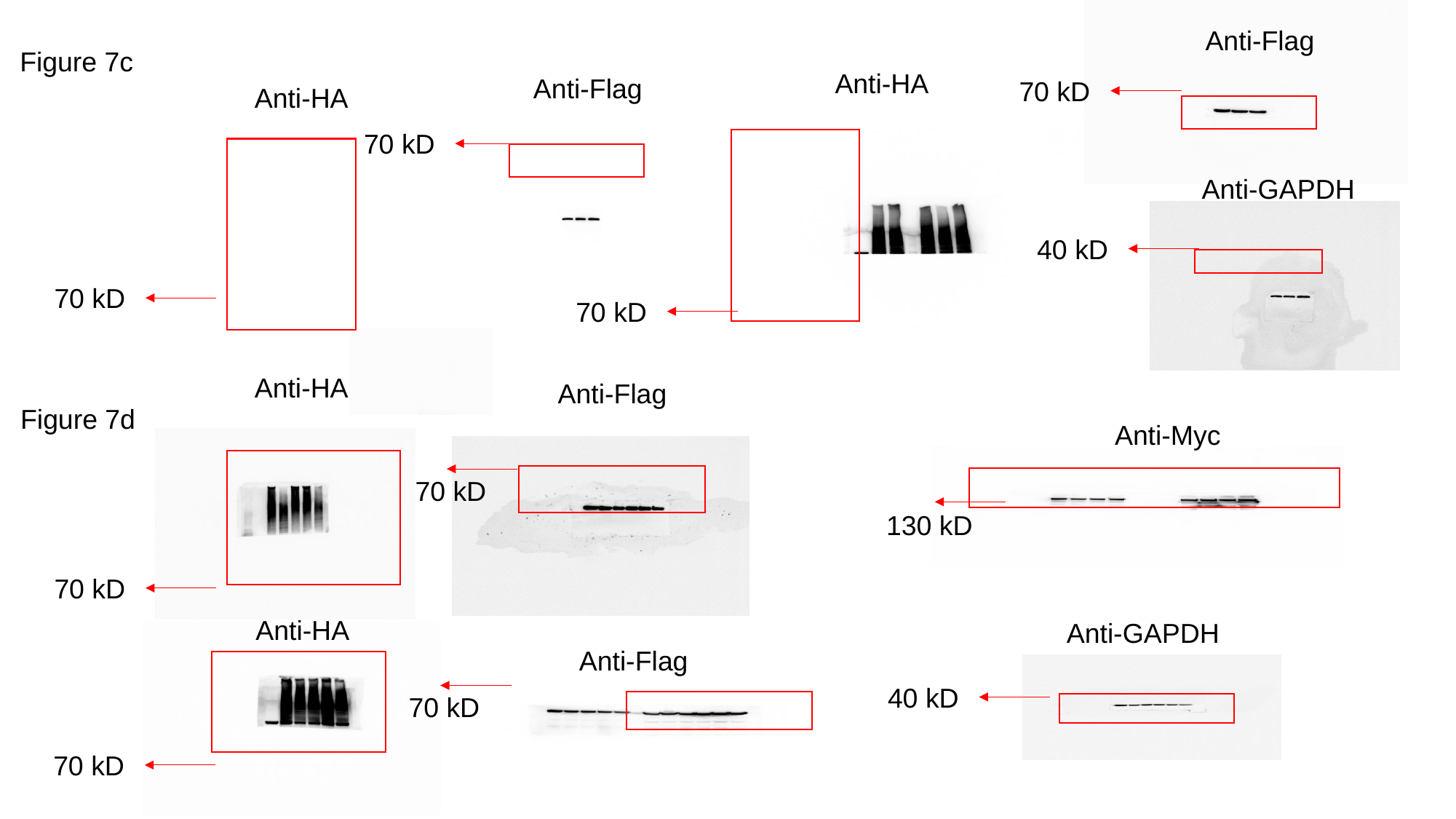

Anti-Flag
Figure 7c
Anti-HA
Anti-Flag
70 kD
Anti-HA
70 kD
Anti-GAPDH
40 kD
70 kD
70 kD
Anti-HA
Anti-Flag
Figure 7d
Anti-Myc
70 kD
130 kD
70 kD
Anti-HA
Anti-GAPDH
Anti-Flag
40 kD
70 kD
70 kD

## Slide 20
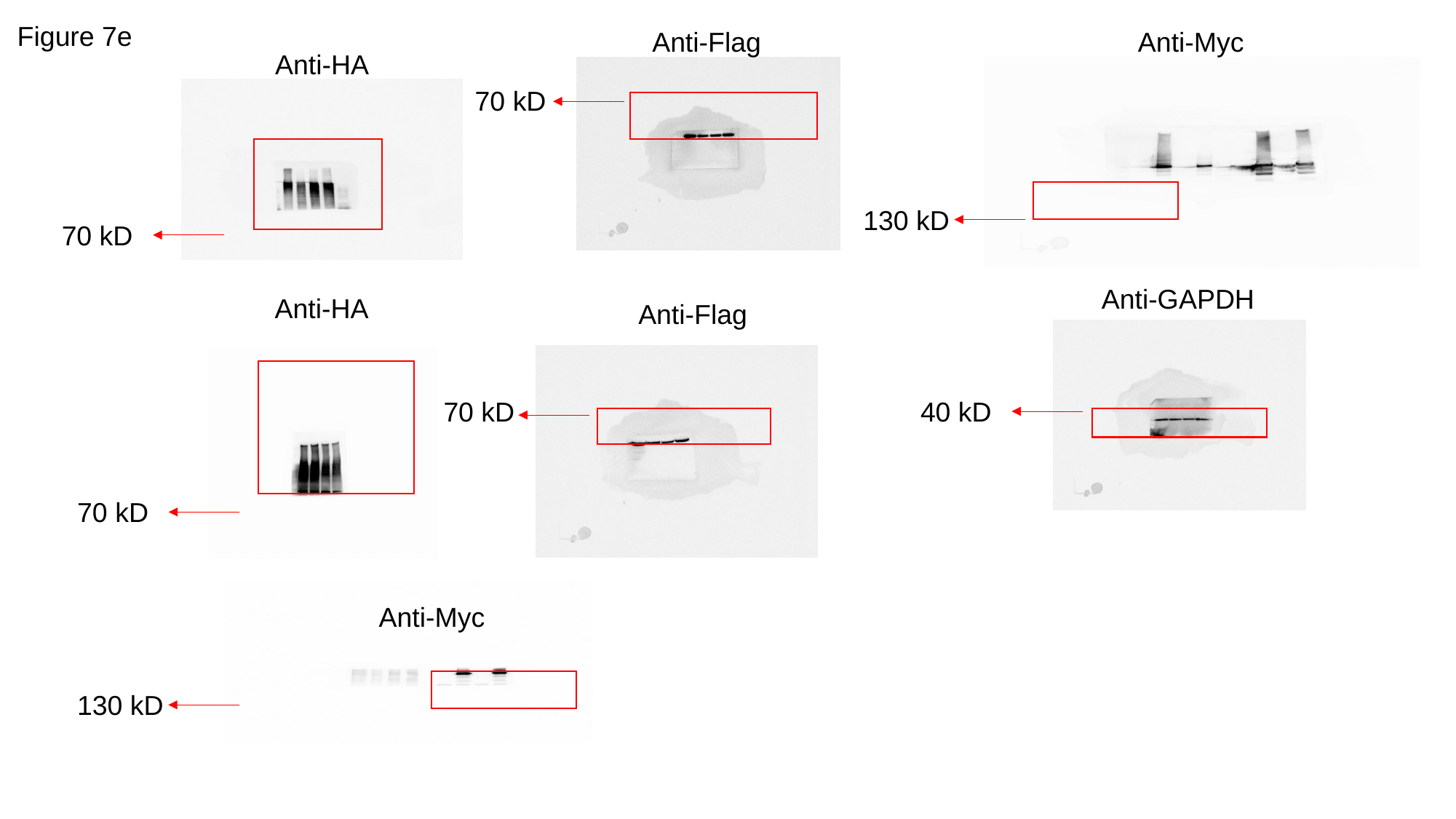

Figure 7e
Anti-Flag
Anti-Myc
Anti-HA
70 kD
130 kD
70 kD
Anti-GAPDH
Anti-HA
Anti-Flag
70 kD
40 kD
70 kD
Anti-Myc
130 kD

## Slide 21
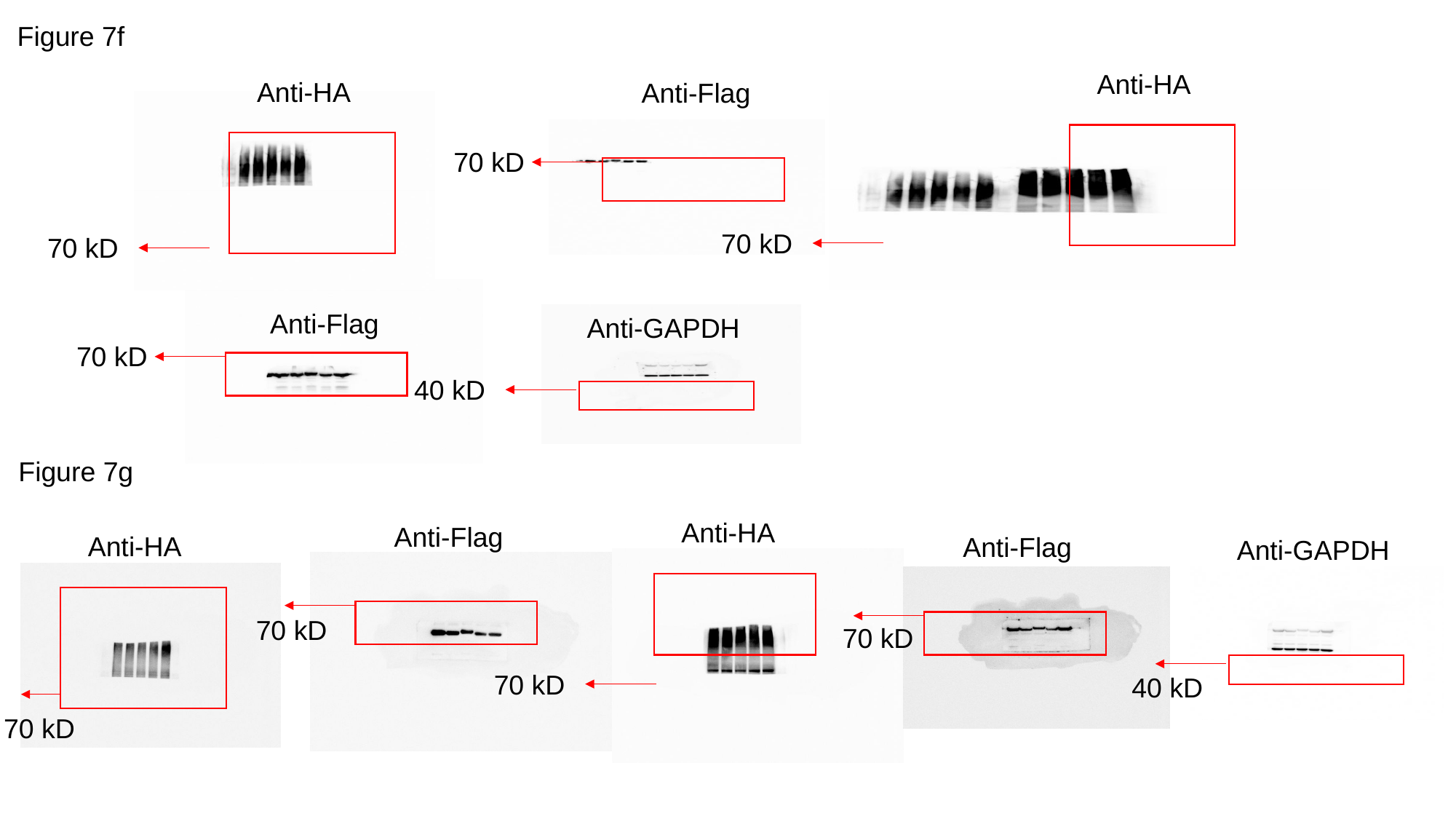

Figure 7f
Anti-HA
Anti-HA
Anti-Flag
70 kD
70 kD
70 kD
Anti-Flag
Anti-GAPDH
70 kD
40 kD
Figure 7g
Anti-HA
Anti-Flag
Anti-HA
Anti-Flag
Anti-GAPDH
70 kD
70 kD
70 kD
40 kD
70 kD

## Slide 22
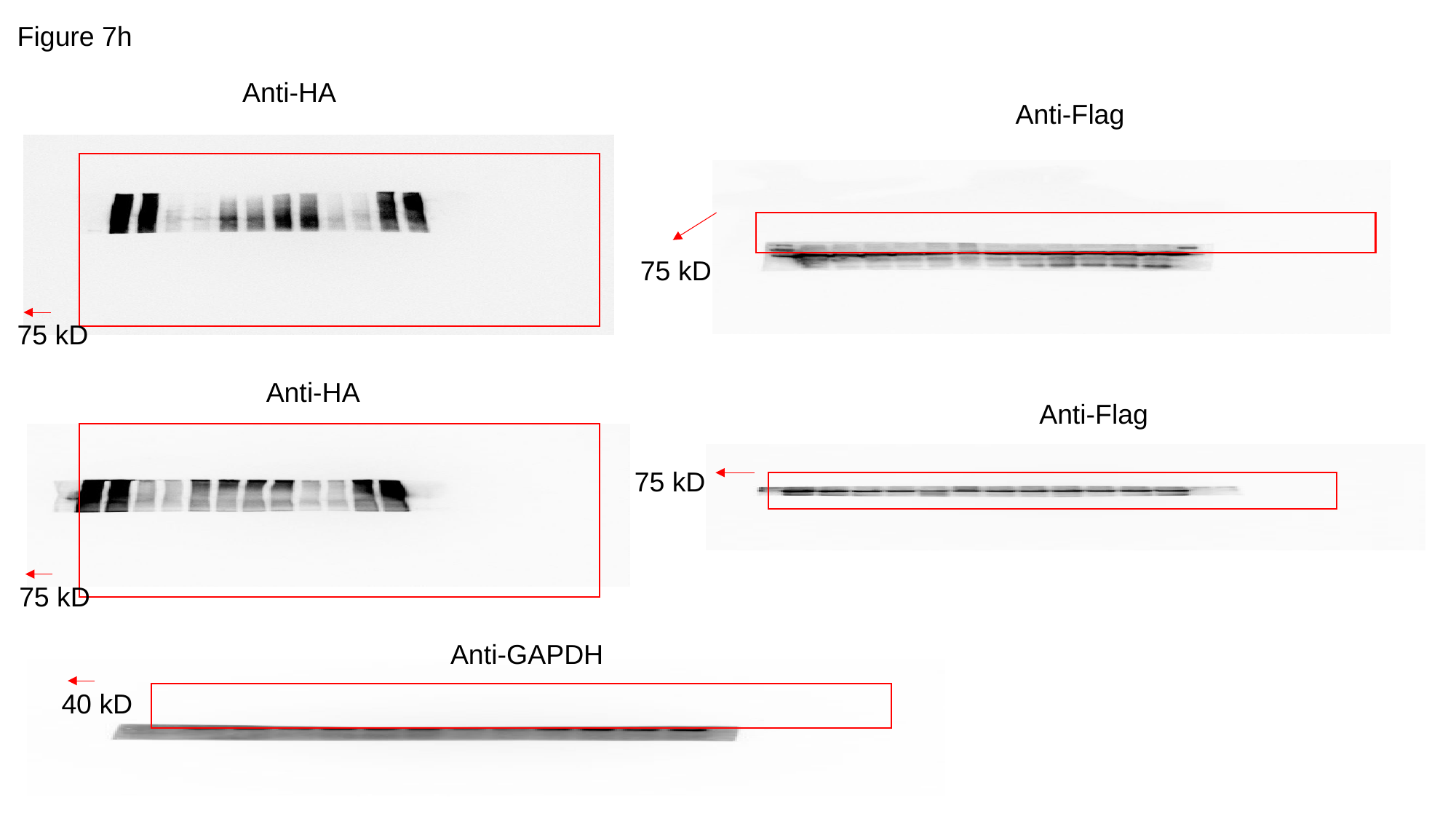

Figure 7h
Anti-HA
Anti-Flag
75 kD
75 kD
Anti-HA
Anti-Flag
75 kD
75 kD
Anti-GAPDH
40 kD

## Slide 23
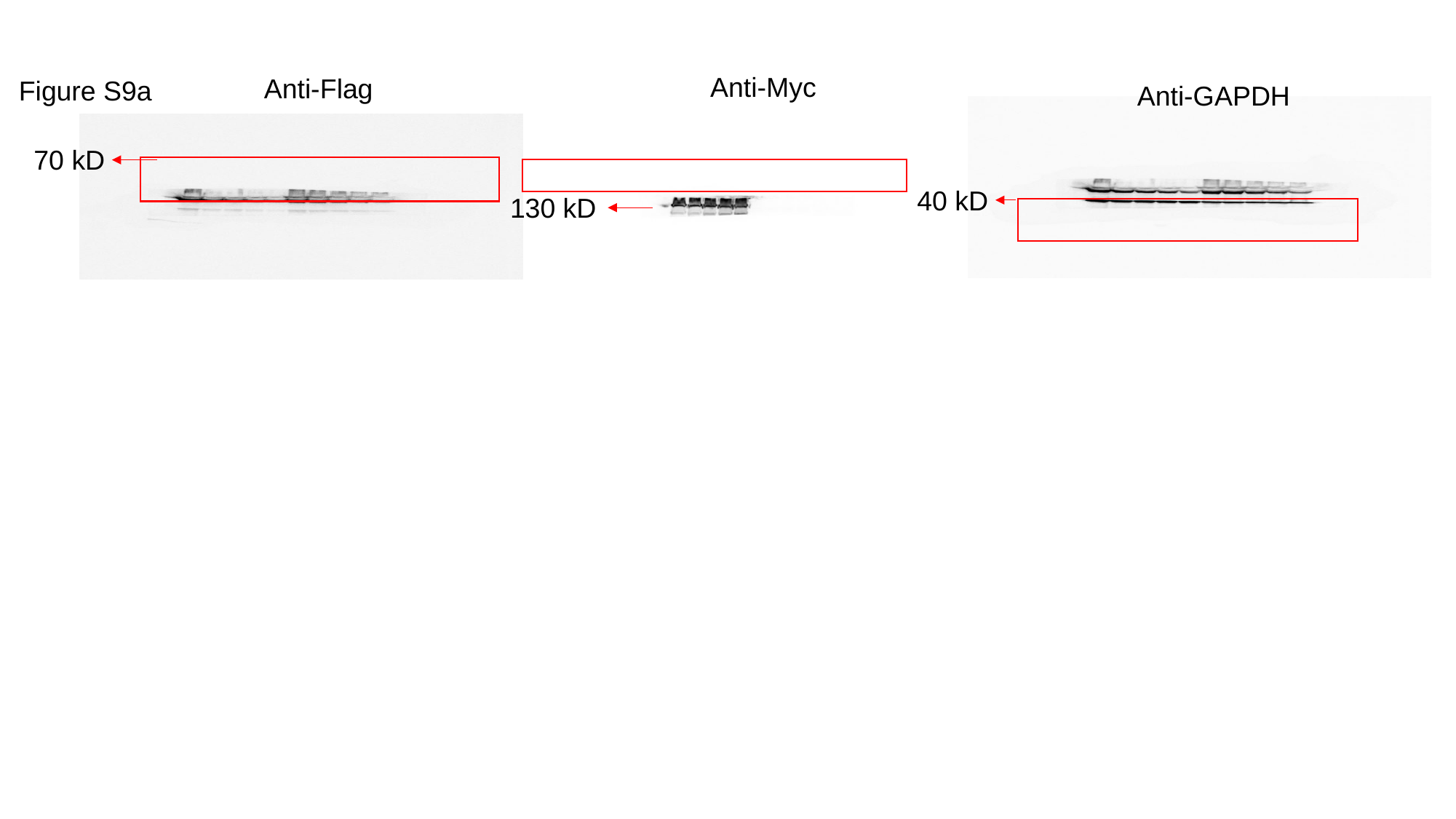

Anti-Myc
Anti-Flag
Figure S9a
Anti-GAPDH
70 kD
40 kD
130 kD
